# Quantitative Treatments for Explaining the Mechanism and Kinetics of Catalytic Electron Transfers in Murburn Processes, Particularly Involving Heme Enzymes Like (Per)oxidases and P450s

**DOI:** 10.1155/bmri/3079294

**Published:** 2026-01-08

**Authors:** Kelath Murali Manoj, Daniel Andrew Gideon, Philip Moses Samuel, Suhotra Das

**Affiliations:** ^1^ Amrita School of Artificial Intelligence, Coimbatore, Amrita Vishwa Vidyapeetham, Amrita Nagar, Ettimadai, Tamil Nadu, India, amrita.edu; ^2^ Satyamjayatu: The Science & Ethics Foundation, Shoranur, Kerala, India; ^3^ Department of Biochemistry, School of Chemical Sciences, St Joseph′s University, Bengaluru, Karnataka, India

**Keywords:** diffusible reactive species (DRS), electron transfer, kinetics, murburn concept, murzyme, xenobiotic metabolism

## Abstract

The seminal Michaelis–Menten theorization for biological catalysis was based on “transition state” (TS), involving the formation of a topologically complementary substrate (S) and enzyme (E) complex (ES) at the “active site” of the latter. Rudolph Marcus put forth the theory of outer sphere electron transfer (ET) in a “donor–acceptor” TS complex, which was seen as a foundational framework for understanding ET reactions in chemical systems. Although these two theories are quite robust, the active site treatment of Michaelis–Menten may not be relevant in promiscuous/nonspecific xenobiotic–metabolizing redox enzymes, and Marcus theory′s applicability to biological ET (BET) systems can be limited in interfacial protein–protein interactions. Herein, the “mathematical” necessity to venture beyond the “active site constraints” of interpreting redox enzyme kinetics and BETs is established first with fresh data. Also, (i) the classical explanation vouching for active site binding and protein–protein complexation–based BET in xenobiotic metabolism (mediated at the endoplasmic reticulum membranes of hepatocytes) and oxidative phosphorylation (multiprotein machinery at mitochondrial cristae) is demonstrated to be untenable, and (ii) tangible/viable murburn models were proposed in lieu. Therefore, toward the imperative goal of arriving at quantitative expressions correlating the parameters/variables involved, the foundational considerations of murburn ET and murzyme catalysis in simple heme systems are presented, with some assumptions/constraints. While some derivations are from ab initio considerations, others are heuristic/empirical, often needing experimental fitting. The linear time‐course profiles of ET (substrate depletion) and the biphasic substrate‐dependent (product formation) are well fit with the newly derived expressions. A mechanistic comparison of the murburn model vis‐à‐vis the longstanding P450cam explanation for drug/xenobiotic metabolism is also provided.

## 1. Introduction: What Is Murburn Concept, and Why Is It Important?

### 1.1. Simple Perception

Imagine a factory where machines do not pass boxes down a fixed assembly line, but instead they are just allowed to float in the air (as there is no one to oversee that they are presented in definite order or sequence), and each tool zips at the boxes as they randomly pass by. Murburn concept says something similar happens in cells, with respect to several reactions. Traditionally, it was perceived that cells work like tight, step‐by‐step machines, especially for energy production, oxygen use, or how drugs are broken down. These old/classical models say everything happens in a strict order on fixed protein chains (at their active sites), like an assembly line (an example being the “electron transport chain (ETC)” of mitochondria or chloroplasts). In a contrasting analogy, the murburn concept offers a new way to look at the affairs of a cell.

“Murburn” is a portmanteau of “*mur*ed *burn*ing” (closed oxidation) or “*m*ild *u*n*r*estricted *burn*ing.” Let us take a paper and burn it. Every time this is done, it burns in a new way, although the effects/products (smoke, heat, flame, ash, etc.) may come out in a similar (but not identical) fashion. With this, it is implied that some reactions in the cell may happen more loosely, with diffusible reactive species (DRS) (exemplified by radicals) floating around. DRS (especially oxygen‐centered radicals) engage in chemical reactions in a controlled yet stochastic manner within the cellular microenvironment, not strictly bound to protein active sites. These DRS can interact with many targets in a delocalized and uncertain manner and still perform useful work without creating a major disorder. It is like controlled sparks in a room full of useful chemicals. As long as it does not spiral out explosively, this can be very efficient and flexible. Murburn concept is not a hypothesis, but it is a “factual theory,” ascertained from the reality that cells are a discretized colloidal soup of interactive molecules (e.g., lipids, carbohydrates, proteins, and nucleic acids) and ions dispersed or solvated in water. Let us see a simple application of murburn, as in the explanation of mitochondrial oxidative phosphorylation (the process which makes ATP, the energy currency of cells). As most physiologies (in)directly depend on this powering process, it is given ultimate priority.

### 1.2. Classical “ETC–Proton Pump‐pmf‐CRAS” Model (Figure 1a)

Figure 1The demarcation between (a) classical and (b) murburn purviews of redox metabolism in mitochondria. While the ETC model works serially sequentially in accounted electron pairs with mobile e‐carriers, the murburn model is explained primarily via 1e‐quilibria within interacting components of the milieu. The ETC model is vitally deterministic (e.g., relatively immobile species like CoQ are supposed to relay electrons from Complexes I and II to Complex III), is thermodynamically untenable, and serves to generate an unverifiable proton gradient. In the murburn model, Complexes I–IV generate/use DRS to catalyze exergonic reactions (via simple bimolecular collisions) to make ATP from ADP bound on them and Pi present at high concentrations in the milieu. The fact that DRS can make ATP is experimentally verifiable.

**Figure:**
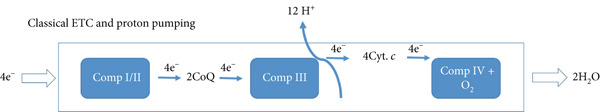
(a)

**Figure:**
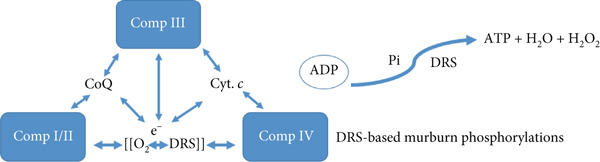
(b)

Stepwise, membrane‐bound complexes (I–IV) pass electrons in a sequential–serial fashion (called ETC) and pump out protons (as per the classical membrane pump theory [CMPT]) to generate proton motive force (*pmf*), which is used to make ATP via a chemiosmotic rotary ATP synthesis (CRAS) mechanism [1–4]. This model is orderly but rigid (and unreal) and fails to explain many physiological aspects like inhibitor promiscuity, cyanide toxicity, thermogenesis, and rapid adaptability. Most importantly, Complexes I through IV could not have evolved deterministically to pump protons in practically aprotic mitochondria [5–8] (and ideas derived from systematic pursuits over 2.5 decades, as presented chronologically in nonoverlapping publications in [9], from Citation #2 to Citation #85 therein!).

### 1.3. Murburn Model Based on DRS (Figure 1b)

Radicals (e.g., superoxide and hydroxyl) form transiently by Complexes I through IV and initiate reactions between ADP (bound at the protein complexes) and Pi in the vicinity of the inner mitochondrial membrane in a delocalized and parallel manner. These reactions are exergonic and do not necessitate any proton pumping or rotary functionalisms, and electrons move via short‐lived diffusible intermediates, not fixed paths. This allows flexibility, thermodynamic viability, and robustness [5–8].

### 1.4. Grass‐Root Level Understanding of Life

With the change in perception above, we can avail totally new ways of interpreting a bevy of molecular processes in cells, as depicted in Table 1.

**Table 1:** A simple overview of the perception changes from classical to the murburn view of biological systems.

**Component**	**Classical view**	**Murburn view**
Oxygen	Final electron acceptor in ETC	Mediator of redox processes/signals
DRS	Considered harmful byproducts	Essential intermediates in moderation
Water (ions)	Dilute solution (solvent)	Discretized colloid & dynamic participant
Enzymes	Rigid active site specificity	Flexible interfaces in DRS flux
Energy flow	Fixed complexes & phase gradients	Spontaneous redox & phase changes
Overall	Irreducibly complex & deterministic	Evolvable & chaos‐ridden (stochastic)

Table 2 gives a listing of the systems/contexts where murburn concept has been applied to avail better explanatory paradigms. The overall holistic outcome is that the umbrella of murburn concept:

i. Explains real‐life flexibility and scope for evolution: Cells deal with many different situations‐new drugs, sudden stress, and strange diets. Murburn shows how cells can adapt quickly, instead of needing a perfect fit every time. The fact that things keep changing gives the scope to evolve.
ii. Disclaims older perceptions and fixes flaws in older models: Some older ideas about energy production (e.g., the ETC) are erroneous. Murburn fills those gaps with a simpler, thermodynamically sound explanation.
iii. Gives new insights into diseases: It may help us better understand cancer, neurodegeneration, aging, drug responses, and why some treatments work differently in different people.
iv. Supports new technology: If we understand how nature uses radicals safely, we could design better bio‐reactors/sensors, therapies, and even smart cells and mainstream some traditional ways of treatments deemed too “unscientific.”

**Table 2:** Examples of diverse molecular and macroscopic systems/aspects of life where murburn concept was applied for explaining outcomes.

**No.**	**Protein system**	**Context/importance**	**Refuted model**	**Can murburn model better explain observations?**	**Author**′**s papers** ^**a**^
1	Heme enzymes (e.g., peroxidases: monomeric soluble enzymes) [This is the pioneering system where murburn concept was established]	Peroxisomes, myeloperoxidase, lignocellulosic, & halogenic organics cycling in ecosystems; detoxification & oxidative stress management	BROS–Compound I (all substrates must bind/access the bound reactive oxygen species at the heme center)	Molecules can get converted even without accessing the distal heme pocket. The protein may liberate DRS, which can interact with the substrate in a delocalized manner. Explains substrate diversity & inhibitions, modulations by diverse molecules, and diffusion‐limited catalysis	*BBA* 2006, *Biochemistry* 2008, *PLoS One* 2010, *BBRC* 2011–2015, *Biochimie* 2016

2	Cyclooxygenase (COX) (membrane‐embedded dimers) & immunology	Inflammation, immune signaling, prostaglandin biosynthesis (pain‐relieving drugs, e.g., the NSAID target!)	BROS–Compound I and Tyr radical react with fatty acids positioned inside protein structure	Explains multiple products and substrate variability. Radical branching and stochastic factors determine the outcome. Fatty acids and inhibitors need not go into protein structure!	*Biomed Rev* 2020, *Biomed Rev* 2024

3	Lactate dehydrogenase (LDH) (soluble tetrameric)	Anaerobic respiration, metabolic shuttling, and insight into cancer metabolism	Reversible reaction of LDH (various isozymes work differently, say in the heart and muscle!)	Explains why lactate must go to the liver or mitochondria to get recycled, in environments rich in DRS! Also, provides awareness of the pH dynamics	*J Cell Physiol* 2022, *AIP Adv* 2023

4	Hemoglobin (Hb) (heterotetrameric soluble protein)	Hb and erythrocytes are some of the most abundant proteins and corpuscles of our body	No equivalents!	Explains prolonged functioning of RBC sans mitochondria and nucleus by pointing out that the tetrameric structure and high‐density packing of Hb are for ATP synthesis and redox balancing. Helps understand Hb glycation	*JBSD* 2022, *AIP Adv* 2023

5	Cytochrome P450 (CYP), its reductase (CPR), and cytochrome *b*_5_ (membrane‐embedded monomers)	Drug/xenobiotic metabolism; detoxification and personalized pharmacology; overall liver functioning and its maladies	P450cam model seeks multimolecular sequential binding and intermolecular long‐distance ET	Explains the clearing of diverse molecules of little evolutionary “context as substrate” and the diversity of CYP with various substrate preferences and modulations and promiscuity of CPR and Cyt. *b*_5_ and overall redox homeostasis	*Front Pharm* 2016, *Curr Drug Metab* 2021, *BBA* 2022, *AIP Adv* 2023

6	Mitochondrial membrane–embedded respiratory proteins (Complexes I to V)	Oxygen‐assisted ATP synthesis and heat generation; the fundamental powering logic of cells that underlines all nonspontaneous works of life	ETC + proton pump + *pmf* + CRAS (highly deterministic gambit‐like logic and multiprotein systems spanning three phases needed to make ATP), fatty acids required to generate heat	Uses the reactivity and mobility of oxygen for a thermodynamically viable redox reaction that explains the structure and distribution of proteins, the kinetics of reactions, and the architecture of mitochondria. The structure of thermogenin matches the predictable function as an interfacial anionic DRS modulator	*ABB* 2018, *Toxicol* 2020, *PBMB* 2021, *BBA* 2022, *AIP Adv* 2023

7	Photosynthesis (light reaction; involves chlorophylls and membrane‐embedded protein complexes)	The tapping of the most fundamental source of energy for life on planet Earth! Logic of optimization of radiation to metabolism	Z‐scheme & Kok–Joliot cycle, LHC as exciton relays to the reaction center of photosystems	Recharts the roles of plastoquinone and plastocyanin, refutes the classical models to explain the diversity of ETs and the overall energetics, gives new reactions, and redefines stoichiometries	*JBSD* 2022a,b, *BA* 2022, *J Cell Physiol* 2023

8	Retinal phototransduction (rhodopsin, transducin, & phosphodiesterase)	Vision is the most primal of our senses. How can light be transduced to a tangible electrochemical signal?	The retinal cycle (11‐cis to all‐trans) has no role for oxygen but only structural changes in proteins	Explains the architecture of the eye, structures of the membrane proteins, and the overall transduction mechanism with light‐assisted reduction of oxygen by rhodopsin and DRS‐mediated phosphorylation of GDP to GTP thereafter	*Biomed Rev* 2020, *J Cell Physiol* 2022, *BBA* 2022, *AIP Adv* 2023

9	Na^+^/K^+^ ion differential & signaling along an axon (membrane proteins, e.g., Na, K‐ATPase, and channels, e.g., KcsA)	How/why do cells have high potassium inside, whereas sodium is higher in plasma? How is a signal relayed along a neuron? How do neurotoxins work?	CMPT (involving cation pumps and selective high‐throughput channels) gives active ways of cellular bioenergetic functioning and intelligence	Refuting ion pumping and larger size–based ion sieving, a thermodynamic and electronic foundation to explain ion differentials and transmembrane potential is in place. The new perspective better explains neurotoxin functions	*J Cell Physiol* 2022a,b, *J Cell Physiol* 2023, *AIP Adv* 2023

10	Bacterial flagellar motility (dozens of radially arranged proteins in the bacterial membrane)	How do bacteria move with respect to chemotaxis using their flagella? (Foundation of mechanical activity/motion and relevant in pathogenesis)	Berg′s rotary model deems that the flagellar basal module functions as a rotor, which enables propulsion through a low Reynolds number regime	Rejecting proton/sodium ion–based rotary principles, water‐ejection propulsion is proposed as the mode for motility. Totally supported by the Type III secretory modular structure and nonconservation of residues	*JBSD* 2022, *AIP Adv* 2023, *Biomed Rev* 2024

11	Unusual dose responses (PDT, hormesis, and idiosyncracies) and integration of parallel systems of medicine	Nonspecific structural outcomes and inexplicable aspects of medical practices observed around the world	No explanations	Has the potential to explain diverse practices and marginally effective theories in parallel systems of medicine and provides the first explanation for hormesis and induction of perturbations in nonchemically connected systems	*Biochimie* 2016, *In Sili Pharm* 2016, *Dose Resp* 2018, *JBSD* 2022, *Biomed Rev* 2024

12	PCHEMS to PTMs; biointelligence to cybernetics; origin, sustenance, termination, evolution of life (OSTEoL); chemicophysical & radiative coherence	Logic of molecular information connectivity, translation to various aspects of systems biology. Can dead systems be revived?	No clear‐cut logic (e.g., PTMs are supposed to be deterministically made by enzymes)	Oxygen–DRS‐centered “noise” is the source of intelligence; it affords scope for connectivity to manmade systems owing to the electronic logic. Perhaps, some dead can be “electronically” revived, with better awareness!	*AIP Adv* 2023, *J Cell Physiol* 2023, *J Cell Physiol* 2024, *Biomed Rev* 2024, *IJMS* 2025

^a^Citation details of KMM′s works can be obtained from [9] or Google Scholar. Most of the above systems are also reviewed in [7, 8].

In short, murburn concept says that cells often use a flexible, DRS‐based system for doing chemical and electromechanical work, not just a fixed step‐by‐step metabolic array of enzymes. It is a fresh, powerful way to understand life′s chemistry, and it could lead to better science and medicine. Murburn concept envisions cellular chemistry not as an assembly line but more like a busy, reactive crowd of molecules (electronic cloud), where radical species freely initiate useful reactions in a controlled environment. It maintains that life thrives not in strict order but in regulated chaos. It is a revolutionary idea with the power to rewrite many core principles in biology.

### 1.5. Current Status on the Murburn Model of Drug/Xenobiotic Metabolism

The murburn model of drug/xenobiotic metabolism was first floated for simple/complex heme–enzyme systems, via poster/oral presentations at the International Conferences on Cytochrome P450s held at Dallas, United States (2005); Bled, Slovenia (2007); and Okinawa, Japan (2009) (Citations #7 to #22 in [9]). The exhaustive evidence/arguments were published for liver‐microsomal cytochrome P450S (CYPs) in 2016 in *Frontiers in Pharmacology,* with 50 comparative points which showed that the murburn model fared better than the P450cam model (Citation #22 in [9]).The research community has shown extreme recalcitrance in changing course, with only sporadic citations of our works in the field [10–14]. A leading researcher in the field of pharmacokinetics was totally dismissive of the murburn model [15], and the pharma industry did not take note either, although the author made repeated attempts to get the concerned involved in debates! In other fields, like the murburn explanations for hormetic dose response [16, 17] and cyanide toxicity [18–22], found more interest in the community. In recent times, the greatest interest and exhaustive discussions were in the field of oxidative stress and mitochondrial metabolism, with some researchers allocating definite sections for the murburn model in reviews [23–27] and book chapters [28, 29], calling for increased attention and discussion on this newly unraveled idea. Murburn concept has also been cited for roles in chromium toxicity–related phosphorylation pathways [28] and thermal reaction efficiency mechanisms [30].

### 1.6. The Agenda of This Work

Given the status of progression on murburn, the next stage, naturally, is the mathematical theorization of murburn concept, as any explanation is better received with quantitative justification/grounding. Soluble peroxidases and liver microsomal and mitochondrial heme–flavin proteins are examples of enzyme systems known to metabolize drugs/xenobiotics [31–33]. It is well documented that these redox systems are also associated with DRS. In this study, we shall explore the basic kinetics of these key enzyme systems and develop novel theoretical approaches to quantitatively underpin the ET catalysis phenomena, along with a comparative mechanistic analysis for explaining the physiological realities and experimental outcomes observed.

## 2. Materials and Methods

This is primarily a theoretical paper that deals with data published earlier, although some new data are also reported (detailed contextually), as per earlier reported protocols.

## 3. Results and Discussion

### 3.1. Classical Treatments for Redox Enzymology and Biological Electron Transfer (BET)

Michaelis and Menten [34] originally derived the equation for biological reaction kinetics based on the “enzyme–substrate complex,” and Briggs and Haldane [35] used the currently standard “steady‐state” assumption for enzyme function in the well‐known textbooks [1–4]. Eyring [36] had developed the “transition state” (TS) (a short‐lived, unstable intermediate of a highly definitive structure) theory for reactions that involved structural changes in the reactants. Marcus [37] built upon Eyring′s work by applying the TS theory to ET reactions between donor–acceptor pairs. Particularly, this was in the case where electrons jump from one atomic/molecular species to another without significant changes in the structure of the reacting molecules but are perceived to do so by merely reorganizing the ambient solvent (which has been traditionally called outer sphere ET). In other words, he formulated an equation that quantifies the barrier for ET, taking into account the energy changes of products–reactants and of solvent reorganization energy. In doing so, he predictably linked the thermodynamics of the ET process to the kinetics of ET. In the theoretical considerations (as discussed above), the treatment was restricted to events occurring at a defined locus of the enzyme–substrate or donor–acceptor pairs, whether in inter‐ or intra‐molecular regimes. Herein, the salient theoretical and experimental premises deemed relevant for us to move beyond the classical paradigms in enzyme‐catalyzed redox reactions and/or BET processes are listed first.

#### 3.1.1. Role(s) of DRS

Marcus′ theory focuses on direct electron transfer in complexes between donor and acceptor molecules. In oxygenated aqueous (biological) systems, DRS (e.g., superoxide, hydroxyl radical, and “hydrated electrons” in respiration/photosynthesis) are often documented in bioenergetic routines and shown to play a significant role in highly endergonic redox reactions and electron transfer processes. These are systematically investigated and documented in the author′s group′s works since 1999 to date [9], which Marcus theory does not explicitly account for. This new angle of exploration could lead to overcoming the limitations in explaining “long‐range intermolecular ETs” in biological systems.

#### 3.1.2. Complexity of Biological Environments

Marcus theory assumes a relatively simple, homogeneous environment, but biological systems are highly complex, microheterogeneous, and dynamic. Proteins, membranes, and cellular structures create intricate microenvironments that can significantly alter electron transfer pathways and rates. Such mosaic solvent effects, ionic strength, and pH variations in biological systems are not always adequately captured by Marcus theory. In nonadiabatic ETs, the donor–acceptor electronic coupling is weak, and the electron can jump between different potential energy surfaces. In such cases, the Marcus activation energy for ET is given by ${\left(\lambda +\Delta G^{{}^{\circ}}\right)}^{2}/4\lambda$, where *λ* is the reorganization energy and *Δ* *G* ^°^ is the standard Gibbs free energy change. Although Marcus theory was extended to adiabatic processes with the Marcus–Hush theory (which introduces a solvent description), it is mostly applicable to nonadiabatic ET (which involves heat exchange between the system and surroundings). In biological systems, some electron transfers can be adiabatic (involve strong coupling with little heat exchange, especially in polarized hydrophobic‐packed redox systems, e.g., those in neurons), whereas some nonadiabatic processes may also feature adjacently. Many biological processes also involve proton‐coupled electron transfers (PCETs). Although Marcus theory can be extended to PCET by considering a second solvent coordinate, it primarily addresses single‐electron transfers and does not involve complex interactive redox equilibria.

#### 3.1.3. Miscellaneous Aspects

Dynamic protein conformational changes and system memory are unaccounted for in Marcus′ theory. Proteins and enzymes in biological systems can undergo conformational changes that affect electron transfer pathways. Marcus theory typically assumes static or averaged geometries, which may not reflect the dynamic nature and mobility of biological molecules. Further, Marcus theory is essentially non‐Markovian (i.e., the past events are accounted in current assessments), the applied equation is in Markovian limits and biological systems have attributes of intelligence/memory. Furthermore, some experimental systems (e.g., that of cytochrome oxidase or Complex IV in mitochondria) had shown only 1/1000 of ET rates in anoxic environments when compared to aerated systems [38], and this did not agree with oxygen′s function (Citation #32 of [9]), questioning the relevance of Marcus model in physiological frames.

### 3.2. Establishing the Quantitative Necessity for a New Approach

Several conclusive fundamental observations/calculations are presented that seek us to go beyond classical treatments:

#### 3.2.1. Peroxidase Hemeprotein System

When heme enzymes like chloroperoxidase (whose functioning is very similar to the myeloperoxidase in blood) are taken at 1–10 × 10^−9^ M concentration (peroxide and chloride at 2 × 10^−3^and 2 × 10^−2^ M concentrations, respectively, and final organic substrate at 10^−4^ M concentration, as seen in Citations #8 of [9]), they give an overall pseudofirst‐order rate of > 100/s (*k*_cat_, number of final substrate molecules converted by one molecule of enzyme in 1 s). It is difficult to imagine this outcome if the overall reactions are solely governed by the active site process. According to the classical Michaelis–Menten mechanistic treatment, this trisubstrate mass–charge minimally balanced reaction would be


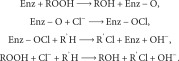


In the above, Enz is the enzyme, Enz‐O and Enz‐OCl are the enzymic intermediates, ROOH is a suitable hydroperoxide, ROH is the “deoxygenated or reduced” product, R’H is the final substrate, and R’Cl is the chlorinated product.

Theoretically and intuitively, the serial/sequentially ordered diffusion of the small peroxide molecule, chloride ion, and the relatively larger organic substrate (which is sometimes even bigger than the active site dimensions!) must take a toll on kinetics. If the diffusion limitation is considered for simple collisions between a large molecule (e.g., protein) and a relatively smaller species (e.g., peroxide or organic substrate and chloride ion), we can consider a second‐order maximal value of 10^8^–10^9^ M^−1^ s^−1^. When this value is multiplied by the participant concentrations, enzyme (10^−8^–10^−9^ M) or final organic substrate (10^−4^ M), it gives a pseudofirst‐order rate, with a minimal 10^−1^–10^1^ s^−1^ (enzyme‐limited) or maximal 10^4^–10^5^ s^−1^ (substrate‐limited) range of values. The above enzyme‐limited constraint cannot stand with experimental observations of pseudofirst‐order ET/catalysis rate approximating ~10^3^ s^−1^ (for an active site excluded substrate!), with classical considerations. Further, for chlorination, the substrate conversion rate was also rather unperturbed by the final substrate type or its concentrations, with the substrate being converted at practically the zeroth order, even under tens of micromolar ranges. An example of the time‐course can be seen in the active site–excluded substrate of thionin (for chlorination), in Figure 2 of Citation #7 of [9]. Please see the profiles in Figure 2b of this manuscript for a comparative schematic. The rate is significantly dependent only upon the enzyme concentration and the ambient presence of supramillimolar chloride ions at acidic pH (and peroxide gave mixed effects).

Figure 2Experimental “time‐course” profiles (increasing time on abscissa and substrate concentration on ordinate axes) observed for substrate depletion or reactant consumption in various biological reactions. (a) Michaelis–Menten enzymes (MM‐enz): curves determined for classical Michaelis–Menten enzyme–catalyzed conversion of substrates (typically, 10–100 nM enzyme and 100–1000 *μ*M substrate). Clearly, the rate lowers dynamically in time because of the diffusion limitations of lowering substrate concentrations. (b) Chloroperoxidase (CPO), a murzyme: Straight lines obtained for chloroperoxidase (CPO)‐mediated chlorination of organics like sulfides and beta‐diketones (typically, 1–10 nM enzyme and 10–100 *μ*M substrate). (c) Microsomal xenobiotic metabolism (mXM) in the liver, a murzyme system: NADPH depletion in xenobiotic metabolizing microsomes. (d) Mitochondrial oxidative (Mito) phosphorylation, a murzyme system: oxygen consumption by respiring mitochondria. The dark/bold arrows depict the depletion of the final substrate, whereas the light arrows show the time point when the final substrate is added. In the profiles of complex systems c & d, it can be seen that the system presents a linear depletion/consumption of e‐source (NADPH) or e‐sink (oxygen), which gets hastened by the addition of final substrate (for say, hydroxylating a xenobiotic RH or phosphorylating ADP).

**Figure:**
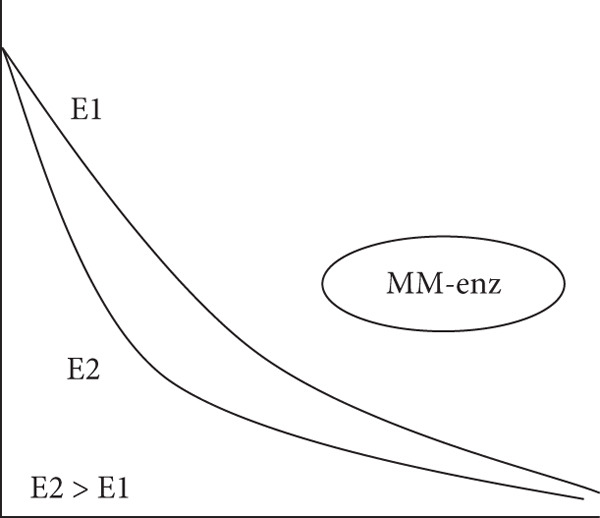
(a)

**Figure:**
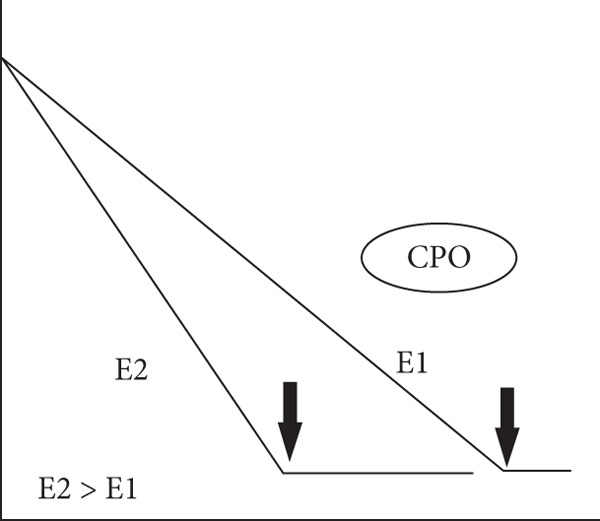
(b)

**Figure:**
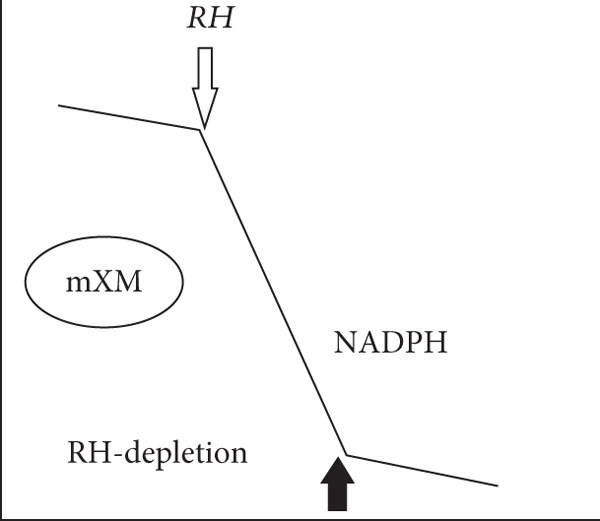
(c)

**Figure:**
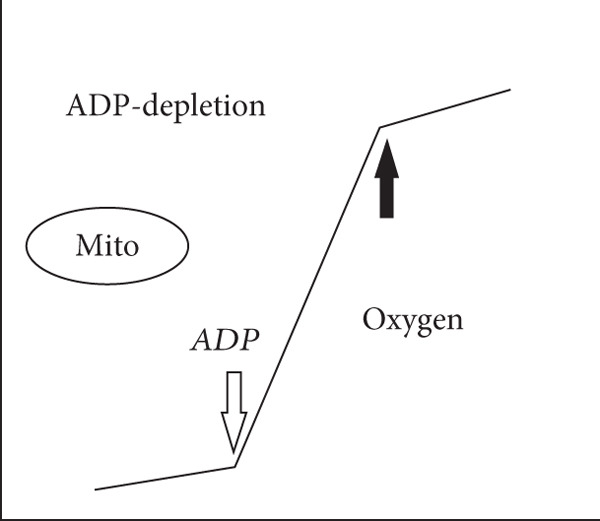
(d)

**Figure 3:**
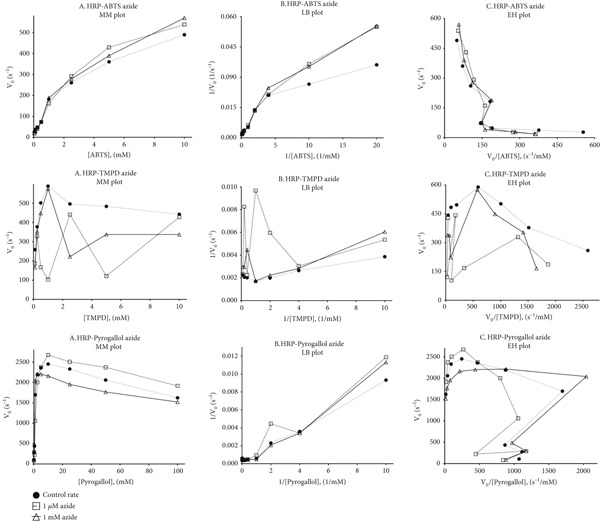
The non–Michaelis–Menten nature of heme–enzyme reactions, as demonstrated by HRP. The first, second, and third rows correspond to ABTS, TMPD, and pyrogallol, respectively, whereas the first, second, and third columns stand for the Michaelis–Menten (hyperbolic?), Lineweaver–Burk (linear plot with positive slope?), and Eadie–Hofstee (linear plot with negative slope?) plots. Conditions were identical to our earlier reported works already cited; [HRP] = 5 nM, [H_2_O_2_] = 2 mM, and KPi pH 7 buffer concentration = 100 mM. The reaction was initiated with the addition of HRP, in a final volume of 1 mL. Azide (an additive that was earlier presumed to be a mere hemeFe ligand and inhibitor!) was included at concentrations of nil (control), 1 *μ*M, and 1 mM. It can be clearly noted that the controls (filled circles) showed lower or higher values than the tests (with low or high concentrations of azide), depending on the substrate type and its initial concentrations. Such observations clearly preclude the adherence of this system to a deterministic/specific Michaelis–Menten kinetics/mechanisms and call for a more nuanced or complex/generic explanation.

**Figure 5:**
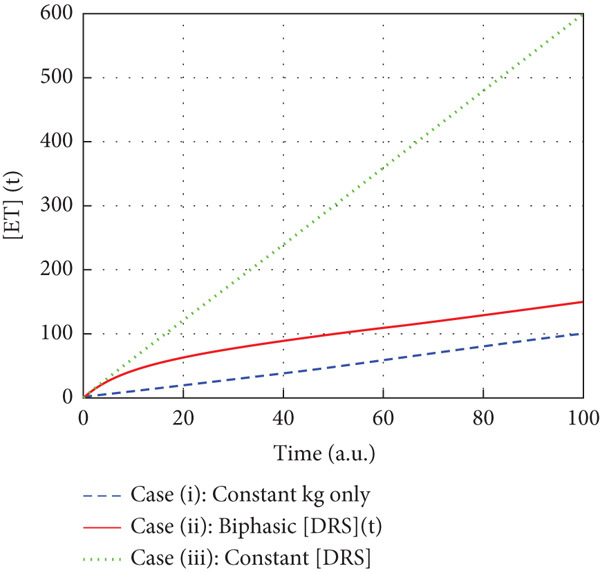
Simulation of electron transfer yields with various treatments under the three theoretical purviews of the murburn model discussed in the text.

Also, when the very same peroxidase enzyme went through active site process, like enantioselective oxygen insertion into a suitable small alkene or organic sulfide molecule, the rates were critically dependent on the alkene substrate concentration, and much higher enzyme concentrations were required to get noticeable conversions (as seen in Figure 2 of Citation #2, Figures 5 and 6 of Citation #4, and Figure 2 of Citation #8 of [9]). These are the typical/usual/classical enzyme–catalyzed curves, which showed significant variations in rates around similar enzyme/substrate concentrations and ratio ranges, as seen in the schematics of Figure 2a. In such cases (Figure 2b), we had successfully theorized that ET/catalysis can only be addressed by the obligatory invocation of reactions in the milieu (outside the active site!) mediated by DRS. The ambiance could stabilize significantly higher amounts of small radicals (DRS) in the milieu at a given instant (say, an order higher than the enzyme concentration), which in turn carried out secondary ET/catalysis outside the active site, and these interactions did not necessarily entail a (re)cycling at the active site. So 8 nM enzyme could give a burst of activity with 80 nM DRS stabilized in the milieu, and this can be multiplied with 10^10^ M^−1^ s^−1^ value for diffusion limitation for smaller species, giving the quantitative scope for explaining the observed experimental pseudofirst‐order rates of 800 s^−1^ for chlorinating conversion of mercaptopurine riboside, explaining the high pseudo‐*k*_cat_ in Citation #8 of [9]. These findings were endorsed by the pioneer in the field, Hager [39], in his lifetime memoir. We reproduce(d) some of the earlier peroxidation kinetics findings in the current work, as shown in the fresh data of Figure 3 and Table 3. From Figure 3, it is easily noted that the substrate‐dependent profile for heme–enzyme catalysis is not hyperbolic. Further, derivative linear plots (Lineweaver–Burk and Eadie–Hofstee) confirm that the Michaelis–Menten relation does not hold good for these reactions. It is highly unlikely that the enzyme has multiple binding sites for such diverse molecules, which influence the enzyme to work differently at varying substrate concentrations! It is more likely that DRS and one‐electron interactive equilibria in the milieu influence the formation and fates of intermediates. Furthermore, an additive like azide (presumed to be a mere inhibitor) can also enhance the product formation in the milieu, serving as a secondary catalyst, working out of the active site regime. The unusual modulatory impact of azide at low concentrations is not via heme binding or any allosteric effects (as demonstrated in Figure 2, Citation #12 of [9]). Table 3 shows a general trend that as the absolute value of enzyme concentration increases (for small phenolic substrates, e.g., pyrogallol, or aromatic amine, e.g., TMPD), the one‐electron abstraction rate gets lowered, owing to competition by other reactions (active site and beyond!).

**Table 3:** Effect of enzyme concentration on absolute value of pseudofirst‐order rate calculations. Conditions were similar to those of Figure 3, except that the concentration of enzyme (HRP) was varied. It can be noted that the rate varies significantly based on changing the concentration of the enzyme, even at low nanomolar ranges, stressing the importance of diverse competing reactions in the milieu.

**HRP (pM)**	**TMPD (s** ^**−1**^ **)**	**Pyrogallol (s** ^**−1**^ **)**
188	2677 ± 149	1951 ± 29
375	1876 ± 21	1853 ± 4
750	1771 ± 1	1671 ± 7
1500	1589 ± 43	1248 ± 0
3000	1567 ± 15	1310 ± 1
6000	1516 ± 17	1414 ± 14
12000	1361 ± 59	1040 ± 6

#### 3.2.2. Hemeprotein P450 + Flavoenzyme System in the Endoplasmic Reticulum

The unusually protracted linearity in heme–enzyme catalysis was also found when the NADPH disappearance rate was monitored in xenobiotic/drug metabolism by CYP microsomes. The profiles of a sample experimental NADPH depletion profile are shown in reconstituted CYP2C9 and CPR controls and reactions (with diclofenac and warfarin as xenobiotic/drug substrates and benzbromarone as inhibitor), with appropriate controls (Figure 4 and Tables 4, 5, and 6). As significant DRS (peroxide) was noted in milieu with CPR alone (without CYP + substrate), the data clearly discount the P450cam model, which necessitates binding of substrate at the CYP′s heme active site for oxygen activation and CPR to CYP ET in such mixtures. Also, the fact that CPR can directly activate oxygen is clearly discerned, and the presence of a substrate with a dissociable proton appears to favor the overall ET from NADPH.

Figure 4Time‐course redox profiles of NADPH depletion in the (a) absence and (b) presence of substrate are shown. (c) Depletion of NADPH is correlated with the generation of peroxide in the milieu in the controls lacking substrate. CPR amounts are more directly correlated to NADPH depletion and DRS production in the milieu. NADPH depletion showed a linear course over time (extending to several tens of minutes, postinitiation) under most scenarios. (The profile on (b) is the same as Nos. 10–12 in Table 6.)

**Figure:**
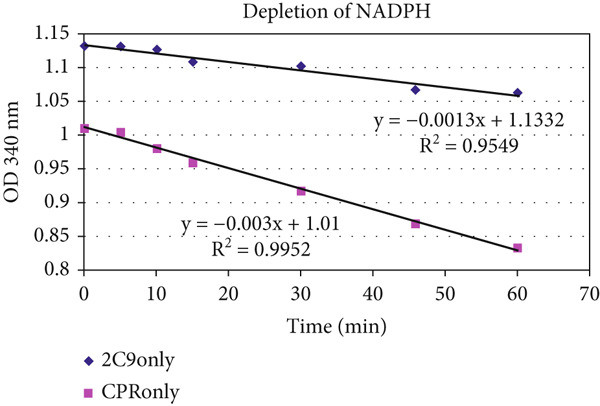
(a)

**Figure:**
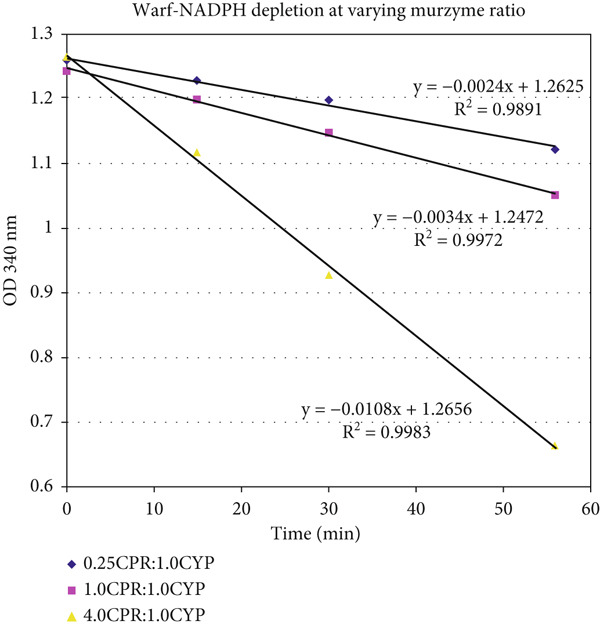
(b)

**Figure:**
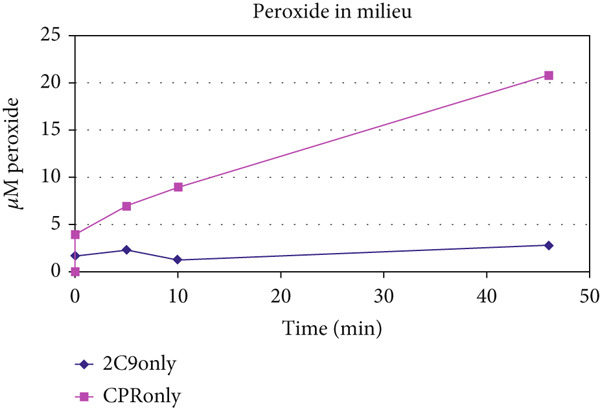
(c)

**Table 4:** Influence of reaction medium (pure and baculosome) and substrate on NADPH depletion; Experiment 1.

**No.**	**System**	**Composition**	**Slope**	**Y** ** intercept**	**R** ^2^
1	Pure reconstituted vesicles	CYP + CPR + Diclof	−0.0136	0.949	1.000
2		CYP + CPR + Warf	−0.0129	1.123	0.996
3		CYP + CPR + BzBr	−0.0127	1.291	0.996
4		CYP + CPR + Diclof+BzBr	−0.0137	1.147	0.997
5		CPR + Diclof	−0.0130	1.031	0.994
6		CYP + Diclof	−0.0007	1.017	0.962
7		CYP + CPR	−0.0126	1.042	0.994

8	Baculosomes (with CYP + CPR)	Diclof	−0.0023	1.105	0.992
9		Warf	−0.0018	1.239	0.980
10		BzBr	−0.0023	1.357	0.981
11		Diclof+BzBr	−0.0025	1.238	0.989
12		Nil	−0.0015	1.115	0.949

**Table 5:** Reactions with various CYP–CPR ratios and controls; Experiment 2.

**No.**	**Description**	**CYP**	**CPR**	**Substrate**	**Slope**	**Y** ** intercept**	**R** ^2^
1	Controls lacking substrate	1	0.2	Nil	−0.0021	1.329	0.997
2		1	0.5	Nil	−0.0028	1.326	1.000
3		1	1	Nil	−0.0044	1.322	1.000

4	Controls lacking CPR	1	0	Diclof	−0.0017	1.304	0.998
5		1	0	Warf	−0.0016	1.402	0.997

6	Controls lacking CYP	0	0.5	Diclof	−0.0012	1.195	1.000
7		0	0.5	Warf	−0.0011	1.292	0.988

8	Control lacking murzymes	0	0	Diclof	−0.0009	1.166	0.998

**Table 6:** Reactions with various CYP–CPR ratios and controls; Experiment 3.

**No.**	**Description**	**CYP**	**CPR**	**Substrate**	**Slope**	**Y** ** intercept**	**R** ^2^
1	Control lacking murzymes	0	0	Nil	−0.0007	0.906	0.944
2	Control lacking CPR	1	0	Nil	−0.0016	0.957	0.992
3	Control lacking CYP	0	1	Nil	−0.0018	0.919	0.957
4	Controls lacking substrates	1	0.25	Nil	−0.0016	1.030	0.978
5		1	1	Nil	−0.0031	1.050	0.989
6		1	4	Nil	−0.0097	1.018	0.995

7	Test reactions with Diclof	1	0.25	Diclof	−0.0025	1.102	0.964
8		1	1	Diclof	−0.0055	1.094	0.996
9		1	4	Diclof	−0.0120	1.124	0.997

10	Test reactions with Warf	1	0.25	Warf	−0.0024	1.263	0.989
11		1	1	Warf	−0.0034	1.247	0.997
12		1	4	Warf	−0.0108	1.266	0.998

In the first part of Table 4, the NADPH depletion profiles in a pure reconstituted CYP2C9 system are tabulated. (Abbreviations: Diclof, diclofenac; Warf, warfarin; BzBr, benzbromarone; CPR, CYP reductase; Baculo, baculosomes [lysed preparations from insect cell lines, with CYP2C9 and CPR optimally coexpressed]. The OD or optical density at 340 nm is at 1 cm path length. Initial NADPH concentration is ~200 *μ*M. For experimental details, please refer to the conditions reported for Figures 2, 3, and 5 in Citation #22 in [9].) The absolute magnitude of the slope increases in the order of (CYP + Diclof) < < (CYP + CPR) ≤ (CYP + CPR + BzBr) ≤ (CYP + CPR + Warf) < (CPR + Diclof) (CYP + CPR + Diclof) ≤ (CYP + CPR + Diclof+BzBr). Clearly, the results are inexplicable with the classical protein–protein complexation ET model. In the second part of Table 4, NADPH depletion profiles in the optimized commercially available baculosomes are shown. The absolute magnitude of the slope increases in the following order: (Baculo only) < (Baculo+Warf) < (Baculo+BzBr) ≈ (Baculo+Diclof) < (Baculo+Diclof+BzBr). Here, the difference in better (diclofenac) substrate and lesser efficient (warfarin) substrate profiles is more marked, but the purported inhibitor (benzbromarone) shows a greater NADPH depletion rate than warfarin, a lesser efficient substrate. NADPH depletion rate is significant even without the xenobiotic substrate, fully negating the classical P450cam model of protein–protein complexation–based ET! The profiles obtained above were confirmed in multiple experiments (for comparable concentrations of the components), as shown in Tables 5 and 6. The clear message is as follows: The enzymes merely sped up the chemical controls and did not bring about any “special effects” on their own merit. That is, controls (lacking the enzymes of CYP or CPR) showed slightly greater NADPH depletion in diclofenac, compared to warfarin, and constitutions with various combinations of the two enzymes merely followed suit. Also, the presence of substrate (not absolutely essential for ET!) merely sped up the DRS dynamics in the milieu (as can be seen from Table 6).

#### 3.2.3. The Mitochondrial System Containing Multiple Heme + Flavin and Other Redox Proteins

Similar to the liver microsomes, in the mitochondrial system, oxygen uptake profiles can be seen to show a linearity phase (correlating with depletion of NADH) from Figure 2d of this manuscript or Figure 19‐18 of Lehninger′s textbook, in the chapter on oxidative phosphorylation [3]. There too, the presence of substrate (ADP + Pi) is the critical factor that displaces the ET equilibrium to the right. The classical treatments fail to explain such anomalies and extended linearities in steady‐state kinetics at depleting reactant scenarios in multisubstrate reactions.

Let us consider the classical enzymology steady‐state treatment, as can be availed in textbooks [40]:

E+S↔ES−−−EP⟶E+P.

The forward and backward rate constants (*k*_1_ and *k*_−1_) leading to the TS complex formation and its reversible breakdown back into the original reactants (*E* and *S*) are related to the Michaelis constant of *K*_*M*_ (the concentration of substrate *S* at which product formation rate *v* is half of the maximal value of *V*_max_, wherein the variables and constants are related by *v* = *V*_max_ · *K*_*M*_/(*K*_*M*_ + *S*)) which is defined as the ratio of net breakdown to formation of TS complex, and it is represented as follows:

(1) KM=k−1+k2k1=Kd+k2/k1.

Here, the constant *k*_2_ pertains to the formation of product from the TS complex, and *K*_*d*_ is the dissociation constant for substrate and enzyme interaction (defined as the concentration at which half of the ligand population remains in the bound state). Therefore, under all scenarios, *K*_*M*_ of the substrate must be greater than its *K*_*d*_. Further, for inhibitions (wherein the assays are carried out by incorporating an extra “inhibiting” molecule in the mixture), the Cheng–Prusoff equation [41] gives the relation of the inhibition indices of the functional *K*_*i*_ calculated from experimental results (it should be theoretically ≈ *K*_*d*_ of the inhibitor determined with pure enzyme and inhibitor binding controls!) and IC_50_ (defined as the concentration of the inhibitor at which we get half the value of functional activity in comparison to the maximal value obtained when the same assay is done without any inhibitor) with *K*_*M*_. The theory notes that IC_50_ and *K*_*i*_ are the same in noncompetitive inhibitions and, also, at [*S*] < < *K*_*M*_ and at *K*_*M*_ < < [S] for the respective scenarios of competitive and uncompetitive inhibitions. But, for the general scenarios of competitive equations, the classical theory dictates that the value of IC_50_ must be higher than *K*_*i*_ for uncompetitive and competitive inhibitions. For the general competitive inhibition scenario, the relation is

(2) IC50=Ki1+S/KM.

Let us now see the data in Table 7 for some ligands with cytochrome oxidase, for the simple case scenario, where Type II binding of the substrate/inhibitor occurs at the active site (depicted by the subscript “as”), via direct coordination to hemeFe, by the following format:

E+S↔EasS;E+I↔EasI.

**Table 7:** Binding parameters of oxygen and inhibitory ligands with cytochrome oxidase. The details of the values are available from compilations made in Citations #35 and #36 of [9].

**No.**	**Measured constants**	**Substrate O**_**2**_	**Inhibitor 1**	**Inhibitor 2**	**Inhibitor 3**
			**CO**	**Cyanide**	**Azide**
1	*k*_on_ [M^−1^ s^−1^]	1 × 10^8^	8 × 10^4^	1 × 10^2^	1 × 10^6^
2	*K*_*d*_ [M]	3 × 10^−4^	4 × 10^−7^	6 × 10^−4^	6 × 10^−5^
3	*K*_*M*_ or *K*_*i*_ [M]	<1 × 10^−7^	3 × 10^−7^	2 × 10^−7^	3 × 10^−5^
4	IC_50_ [M]	**NA**	4 × 10^−5^	5 × 10^−5^	7 × 10^−7^
	Derived constant				
5	*k*_off_ (from 1 & 2) [s^−1^/Hz]	**30000**	0.003	**0.06**	**0.6**

*Note:* The values in the third (for oxygen) and fifth (for cyanide) columns are in boldface for easier comparison.

Some stark violations of classical theoretical mandates are evident!

1. The equilibrium dissociation constant *K*_*d*_ of oxygen is about 3000‐fold higher than the functional activity coefficient *K*_*M*_ of oxygen, which voids Equation (1).
2. Experimentally calculated *K*_*i*_ values of the inhibitors are lower (surprisingly!) than the corresponding molecules′ directly measured *K*_*d*_, and this is more pronounced in the case of cyanide, which directly voids Equation (2) (and 1 too!).
3. IC_50_ values of cyanide and azide are lower than their corresponding *K*_*d*_ values, which again voids Equation (2).
4. IC_50_ value of azide is lower than its *K*_*i*_ value, which yet again voids Equation (2). Therefore, the Michaelis–Menten theorization for oxygen/ligand binding–based interactions is inadequate to explain the outcomes in physiology, in the above context.

The gram molecular/ionic weight of the diatomic species of oxygen, CO, and cyanide is comparable: 32, 28, and 26, respectively; they are of similar mass and size. The binding‐based explanations cannot explain the acute toxicity of low doses of cyanide when considering that

1. The thermodynamic dissociation constant of oxygen for cytochrome oxidase is half that of cyanide, and oxygen concentrations (9 mg/L or~300 *μ*M) are higher or comparable to the toxic dose of cyanide (~3 mg/L or ~100 *μ*M) in blood! This means that oxygen could effectively outcompete cyanide for hemeFe binding in lethal scenarios.
2. The amounts of heme proteins in blood/cells far exceed the lethal amounts of cyanide by a ratio of 100:1 or 1000:1, negating the stoichiometric binding–based accounting/outcomes. That is, even if an ion of cyanide outcompetes oxygen and binds with 100% efficiency, it would still leave behind 99%–99.9% of hemeproteins fully capable of function.
3. The experimentally determined *K*_*d*_ of CO–hemeFe is 1500‐fold lower than cyanide–hemeFe. The *k*_on_ ratio of CO and cyanide is 800 (carbon monoxide binds faster than cyanide), and the *k*_off_ ratio of cyanide and CO is 20 (carbon monoxide–hemeFe complex dissociates less frequently than cyanide–hemeFe complex). In compounded terms (thermodynamically cum kinetically), CO is ~24 million‐fold (1500 × 800 × 20) more efficient ligand for hemeFe than cyanide!! The binding‐based explanation, therefore, totally fails to explain why more than 200 mg/L (~7000 *μ*M) of CO is the lethal dosage (wherein loss of consciousness and death occur slowly in CO poisoning!), whereas ~300 *μ*M cyanide knocks out and kills instantly. For more detailed mathematical argumentation and data regarding the mechanistically crucial cyanide‐based inhibition of heme proteins, please check Citations #17, #19, #21, #35, and #36 of [9].
4. In a connected explorative work, we had shown that another similar ion, azide (taken at low concentrations), considered a hemeFe binding inhibitor (quite like cyanide), actually enhanced several heme–enzyme catalyzed peroxidative electron abstraction reactions by many folds (#12 citation of [9]), and this is also shown in Figure 3 of the current work! This is yet again an anomaly that the binding‐based classical explanations could not explain.

#### 3.2.4. Miscellaneous Findings

This paragraph exclusively deals with some of our findings, as listed/cited in Reference [9]. Via Table 3 of #46 and Table 2 in #54, it was shown that Marcus equation′s outer sphere ET treatments were inadequate to explain the qualitative and quantitative outcomes (thermodynamic feasibility and kinetic viability) observed in several bioenergetic redox reaction steps that entailed unfavorable distances and redox potentials, particularly in intermolecular contexts. Further, the analysis of structures (particularly, the solvent‐accessible redox centers), ET kinetics, and promiscuity of small redox proteins like cytochrome *c*, cytochrome *b*_5_, plastocyanin, and ferredoxin (Citations #15, #16, #41, and #44) clearly showed that protein–protein complexation–based proposals were not viable, and a more generic mechanism was operative. Furthermore, with the unusual profiles of activity (Citation #9), the ability of trace amounts of a molecule to enhance activity (Citation #13), diversity of modulations (several different instances of a given molecule being an activator and inhibitor at different concentrations; Citation #20), multiple substrates showing concentration‐dependent inhibition kinetics for the same enzyme (Citation #10), and hormetic dose responses (Citation #24) in multitudes of heme–enzyme/substrate/modulator systems, the mandate for thinking beyond the Michaelis–Menten kinetics was clearly established. Therein, it was experimentally demonstrated and/or theoretically inferred that classically determined *K*_*M*_ or *K*_*i*_ or IC_50_ values did not show adherence to the typical Michaelis–Menten norms (Citation #17). Venturing beyond the classical “enzyme–substrate complex” of enzyme function or “transition state” theory of “donor–acceptor complex” formation in ET (deemed to occur via a deterministically unique and topologically complemented transient intermediary structure), DRS‐based murburn postulation explained the outcomes and the structural/architectural features of the membrane proteins, organelles, and cells involved (Citation #57). It was deemed that some reactions proceeded via a rather “multiroute stochastic” process, often involving DRS, which could invoke interactive dynamics with diverse components in the milieu.

#### 3.2.5. Summation

Herein, it is clearly affirmed that we do not negate the veracity of the Michaelis–Menten or Marcus theories; it is just claimed that some enzymes (as exemplified by heme enzymes like peroxidases and P450s) work in a different way! In this context, murburn concept emphasizes the role of DRS and stochastic interactions, which can be more aligned with the complexity of biological environments. Here is why the murburn theorization may be more appealing in certain contexts:

##### 3.2.5.1. Explicit Attention to Effective Charge Separation (ECS) Leading to the Production/Activity of DRS

The murburn model highlights the importance of diffusible radicals and DRS/reactive oxygen species (ROS) in mediating electron transfers, which is particularly relevant in biological systems where ROS are often involved in signaling and metabolic processes. It posits that the kinetics would be dependent on the in situ concentration and availability of DRS, and herein, a linear correlation of rates may or may not derived with respect to DRS. The outcome would be contingent upon the *generation*, *diffusion*, and *lifetime of reactive intermediates* (e.g., superoxide and hydroxyl radicals). DRS availability also depends on local environmental factors like oxygen concentration, redox state, solvent organization, pH, temperature, types of ions, and ionic strength.

##### 3.2.5.2. Stochastic and Nonspecific Interactions Explain Several Complex/Unconventional Observations

Unlike Marcus theory, which assumes specific donor–acceptor pairs, the murburn model accounts for stochastic, nonspecific interactions between molecules. This is more reflective of the chaotic and crowded nature of cellular environments. The murburn approach has explained phenomena in complex, multicomponent systems like mitochondria and chloroplasts, where traditional theories like the Michaelis–Menten kinetics and the Marcus ET model fall short. The explanations for idiosyncratic and hormetic physiological dose responses are other examples of success stories of the murburn concept in biology. Unlike classical enzyme–substrate interactions, murzyme ET/catalysis could be nonselective and nonspecific, with DRS diffusing freely and interacting with targets in a probabilistic manner. Therefore, the environmental conditions and concentration/reactivity of all of the milieu components could be more directly important than they are in the classical scenario.

##### 3.2.5.3. Direct Justification of Thermodynamics/Kinetics/Probability and Providing New Dimensions to Cell Biology

Murburn models satisfy thermodynamics, kinetics, and probabilistic considerations at all levels and integrate them in a way that is more adaptable to the dynamic and nonequilibrium conditions often found in biological systems. In doing so, it also affords a broader scope for redox enzymology/biology that extends beyond electron transfer to encompass and impact epigenetics, posttranslational modifications, cellular phase changes, signal transductions and cascades, and pathology. Reactions involve zeroth, first‐ or second‐order kinetics depending on the concentrations/nature of the DRS and the substrate (and environment). The dynamic interplay between *enzyme*, *substrate*, *milieu components*, and *radicals* is crucial. The generation rate of radicals also vary based on the environmental features mentioned earlier and the presence/concentration of antioxidants and inhibitors.

### 3.3. Theorization of the Quantitative Aspects of Murburn Processes

The stochastic or probabilistic nature of murburn reactions must be considered with (i) the theory of free radical reactions, (ii) quantum mechanical approaches for redox‐active systems where electron tunneling and radical coupling are significant, and (iii) systems biology models wherein incorporating environment‐dependent variables like diffusion and stochastic interactions would be considered. Experimentally, substrate depletion or product formation over time would need to be determined. In light of the key factors mentioned above, the theorization is as follows.

#### 3.3.1. Minimal Treatment for Net ETs Observed

A general equation that captures the key principles of DRS, stochastic interactions, and dynamically evolving scenarios is proposed. The overall ET would be contingent upon a minimum of three overlapping (not necessarily mutually exclusive, in a strict functional sense!) processes:

Overall ET=generation of DRS+loss/buffering of DRS+ET to the target.

In the first term, the e‐source (e.g., NADH/NADPH or peroxide or any other suitable electron‐rich molecule of favorable redox potential) and murzyme (Enz; the latter could regain the lost electron from one of its substrates!) or oxidant (Oxi) could interact to generate the DRS. Examples of such processes include the following: A flavoprotein could activate oxygen, or a reduced oxygenated hemoprotein could dissociate to give superoxide. In the second term, the generated DRS can be transiently or permanently lost owing to competitive reactions with the milieu components (*M*) therein. For example, the one‐electron active superoxide above can alter redox states of suitable e‐sponging murzymes like cytochrome *c* or get dismutated to peroxide and water thereafter (if DRS is present in adequate amounts and protons are non‐limiting). The final term is the observable outcome or the reaction of interest wherein the DRS interacts with a protein or small molecule (Tar, the final target!), thereby leading to effective e‐sinking, enabling the e‐flow within the system to chug along once again from source to sink. Expressing the overall event(s) in temporal rate terms

dETdt=Σkg·e‐source·Enz or Oxi+ΣkComp·DRS·M+ΣkReact·DRS or Enz∗·Tar.

In the above, *Σ* represents the sum of all discrete events leading to DRS generation, DRS competition, and fruitful DRS reaction, respectively; and *k*_*g*_, *k*_Comp_, and *k*_React_ are rate constants for various reactions under these three respective heads (generation of DRS, competitive loss or sequestration of DRS, and reactive utility of DRS). As there could be multiple distinct species for most variables listed in the equation above (*e*‐source, Oxi, DRS, *M*, and Enz∗), the respective constants would also vary thereof. It is evident that the equation involves multiple variables (*e*‐source, Oxi, DRS, *M*, Enz/Enz∗, and Tar) that are not mutually independent, and their temporal dynamics are neither linear nor unidirectional. Further, each term in the sums represents a distinct reaction pathway, and the rate constant may differ for each case. For a more accurate and general solution, numerical integration or a more detailed model accounting for the interdependence of species and nonlinearities would be necessary. However, to simplify (with practical relevance), we can consider an assumption wherein (i) initial rate measurement conditions are such that the concentrations of components are nonlimiting and (ii) multiple pathways are clumped into common rate constants. Then

(3) dETdt=kg+kComp·DRS+kReact·DRS,dETdt=kg+kComp+kReact·DRS.

If we take the above as the fundamental governing scenario for ET in murzyme mixtures, we can consider three minimal scenarios.

i. We can consider a condition where the absolute value of [DRS] or (*k*_Comp_ + *k*_React_) is minuscule compared to *k*_*g*_. Then, we have
dETdt=kg.

Under the above simplifying assumptions, the integrated solution is

(4) ETt=ET0+kg·t.

It predicts a linear increase in electron transfer over time (where [ET]_0_ is the net ET at the initial time).

ii. If we consider a time‐dependent [DRS] value (by virtue of, say, an exponential function that reaches an asymptote), then we have the following:
DRSt=DRS0·e−λt,dETdt=kg+kComp+kReact·DRS0·e−λt.

Then, upon integration

(5) ETt=ET0+kg·t+kComp+kReact·DRS0/λ·1‐e−λt.

Under this assumption, the integrated solution predicts a biphasic ET accumulation, with an initial burst due to [DRS] and a long‐term linear growth due to steady *k*_*g*_.

iii. If we assume that *d*[DRS]/dt = 0 (either we have practically no DRS in the milieu or there is a steady production and depletion of DRS, and its concentration in the milieu remains constant), then Equation (3) becomes
dETdt=keff,where keff=kg+kComp+kReact.

Upon integration, this too takes the form of the first case scenario:

(6) ETt=ET0+keff·t.

We shall have a linear increase in electron transfer over time, but the slope will be different from the first case. If [DRS] is nonzero and constant, its value will be higher than in the first case scenario. If [DRS] = 0, then the case is the same as the first one.

In all three cases, we can avail the premise within the optimized murzyme‐mediated ET cases (as seen/shown in the schematic time‐course profiles of the three cases of Figure 2: single‐enzyme chloroperoxidase–mediated chlorination, bi‐enzyme system CYP reductase for xenobiotic‐metabolizing microsomes, and multienzyme system of mitochondrial oxidative phosphorylation) wherein the linear phase of ET transfers is protracted, supporting the murburn (DRS‐mediated) theorization of ET. This is quite unlike the MM theorization, wherein linearity is practically nonexistent (as the rate changes quickly in time because of binding requirements, which reflects in the decay of ET in time, as shown in Figure 2a). A simulation of murburn ET kinetics for the three case scenarios discussed above is shown in Figure 5, and the corresponding MATLAB code is given in Box 1 (Supporting Information (available here)). The slope change (rate enhancement) in experimental plots in complex heme–enzyme systems (Figure 2c,d) by the “thermodynamic pull” exerted by the e‐sinking substrate can be reasoned out by the shifting of reaction milieu from Cases (i) and/or (ii) to Case (iii) of the theorization discussed above and as simulated in Figure 5.

#### 3.3.2. For Simple Enzyme Catalysis (Say, 1e Abstraction) Profile Leading to a Specific Product

The simple case scenario for a heme enzyme (e.g., chloroperoxidase) can be captured with the following reaction steps:

1. Murzyme + oxygen and/or peroxide (2e − stable activators) = murzyme∗+DRS
2. DRS competitions with milieu components (enzyme/molecular/ionic species) = diverse outcomes
3. DRS or murzyme∗or activated milieu species + substrate = product(s)
4. DRS or murzyme∗or activated milieu species + product(s) = alternate product(s)

Based on such a theorization, the kinetics for overall one‐electron abstraction from a substrate to give a specific product mediated by the murzyme could be modeled under various heads as

1. DRS generation rate = *k*_*g*DRS_ [murzyme] [oxygen and/or peroxide]
2. Internal competitions = *k*_1_ [DRS]^*m*^; *k*_2_ [DRS] [Comp1]; *k*_3_ [DRS] [Comp2]; *k*_4_ [DRS] [*X* ^−^]⋯
3. Substrate conversion rate = *k*_1sub_ [DRS] [Sub] + *k*_2sub_ [Comp1] [Sub] + *k*_3sub_ [Comp2] [Sub] + *k*_4sub_ [*X*∗] [Sub]⋯
4. Product depletion rate = *k*_1prod_ [DRS] [Prod] + *k*_2prod_ [Comp1] [Prod] + *k*_3prod_ [Comp2] [Prod] + *k*_4prod_ [*X*∗] [Prod]⋯

(7) OveralldPdt=DRSk1subSub±k1prodProd+Comp1k2subSub±k2prodProd+Comp2k3subSub±k3prodProd+X∗k4subSub±k4prodProd⋯

Please see that in the first step of derivation above, murzyme need not be essential, and if spin states permit, activating molecules could get altered directly with a suitable electron‐rich or electron‐deficient molecule in the milieu, which could thereafter also involve murzyme cycling. (In multiprotein and multiactivator systems, the scenario gets even more complex! Such discussions are beyond the context of the current article and will be focused upon in a forthcoming communication.) The summary of the last equation above (for *d*[*P*]/dt) is that it is a sum of many terms, with each term consisting of a rate constant *k*, substrate concentration [Sub], product concentration [Prod], and a multiplying factor ([DRS], [Comp1], etc.). The overall reaction scheme′s fate is subject to high levels of variation. Also, the initiation of the reaction need not be in any particular serial/sequential scheme, as the outcomes are contingent upon fluctuating 1e/2e redox equilibria in the milieu.

The equation is not as simple or elegant with only one or two constants, as the one derived in, say, the Michaelis–Menten kinetics for a hyperbolic asymptote. While it is not facile to simplify the above theorization further (owing to the uncertainty involved in DRS/product formation/depletion dynamics), the equations could be incorporated into a sophisticated mathematical model and computed to capture the functional essence (the various constants) of the overall system, starting from a given set of concentrations. Only the complex equation above can explain the multiphasic nature and complexity of time‐course and substrate‐dependent curves for product formation.

When listed for the total terms, the equation above can be integrated (with some simplifications), to give the product at a definite time, *t*. Currently, we have limited experimental modalities of dealing with the complexities involved (although online mass spectrometry could provide some insight into the dynamic tracing of the fates of initial materials and intermediates within the reaction milieu) to determine the various constants in the equation above. However, the variables and constants can be easily addressed as such by the currently available AI methodology. Experimentally, the product observation rate could be landscaped for the relevant concentration of variables, and the comprehensive data obtained (by changing the variables with respect to each other) could be availed to enable a numerical solving (not analytical simplification) of the equation above. At low enzyme/reactants′ concentrations, the contributions of Comp1 and Comp2 (Compounds I and II, respectively, wherein the enzyme is at higher oxidation states than the native Fe(III); it is also known that there are enzymic species with lower oxidation states, and these could also interact with substrate and DRS!) would be low and that of DRS/X^∗^ would be high, and at higher *E* and *S* concentrations, the latter (bound reactive oxygen species [BROS]) would prevail, and DROS lifetimes would be minimized. The constitutive controls in such a catalytic system would be as follows (in no particular order): dynamic partitioning of reactants, products and intermediates, dielectrics of the reaction ambiance, concentration effects, proton availability, spin controls (of the metal and oxygen species), and charge transfers and stabilizations of the metal species (e.g., Fe II vs. Fe IV). (It is pertinent to iterate that the high kinetics seen in nonenantioselective reactions involving nM enzyme and *μ*M substrate are mechanistically distinct/different from the enantioselective reactions involving *μ*M enzyme and mM substrate. The “synthetic‐scale mixtures” of the latter type [which are also scaled akin to spectroscopic/crystallographic/cryomicroscopic preparations] have high ligand pressures and high absolute concentration of active sites, whereby the ligand may achieve active site binding in quite a facile manner, which cannot be the case under the routine dilute concentrations taken for enzyme assay.)

Now, for empirical rate calculations/treatments and simplification, we can view the reactions under the following considerations.

If we assume that all terms contributing to the reactions in Equation (7) sum up to an effective rate constant *k*_eff_ and that substrate consumption follows first‐order kinetics, then

dPdt=keffSub,

where

keff=∑sum of all iki·react.spec.i,

then

dSubdt=−keff·Sub.

Upon integrating, we get

Subt=Sub0·e−keff·t.

Since [*P*] is formed at the expense of substrate depletion, we get

Pt=Sub0−Subt.

Substituting for [Sub](*t*) and simplifying, we get

Pt=Sub0−Sub0·e−keff·t,

(8) Pt=Sub0·1‐e−keff·t.

The starting‐point differential equation accounts for multicomponent cascade with various sources of DRS, competing reactions, and substrate–product conversions. Under steady‐state assumptions, the summation over multiple kinetic terms introduces the possibility of minimizing fluctuating rates due to variable DRS contributions. Such a summing of all terms converges to an effective rate constant *k*_eff_, wherein a pseudofirst‐order approximation emerges (with a largely unique rate‐limiting step, at least in the initial phase), with a dominant term which governs the kinetics, leading to a clean exponential profile.

However, in several murburn mixtures with electron‐rich substrates (with activated nucleophilic reaction centers), the catalysis rate is experimentally observed to be correlated more with the initial enzyme concentration (or dynamic [DRS]) alone. In such scenarios, the temporal dynamics of DRS can be represented minimally as

dDRSdt=kgDRS−kdecay·DRS,

which, when integrated, gives a simple exponential decay:

DRSt=DRS0·e−kdecay·t,

where [DRS]_0_ is the concentration of DRS formed/stabilized at the initial time. The concentration of DRS itself can be envisaged to be a temporal function, and many milieu reactions involving multiple exponential dependencies contain *feedback*, *self-organization*, and *autocatalysis means*, many times leading to nonlinear kinetics. (But the above treatment is only a simplified version.) In scenarios where the rate is practically the zeroth order with respect to the substrate, we have

dPdt=keff·DRS.

To find the product at time *t*, we need to integrate this, as follows:

ʃdP=ʃkeff·DRSdt.

Substituting for [DRS] in the integral

ʃdP=ʃkeff·DRS0·e−kdecay·tdt,Pt=keff·DRS0/kdecay·1‐e−keff·t.

Therefore, for empirical/practical reasons (at nonlimiting electron‐rich substrate concentrations), we can convert the term of (*k*_eff_ · [DRS]_0/_ *k*_decay_) in the equation above into “*k* · [Enz]_0_,” to signify that the burst of DRS is a function of initial enzyme concentration and a constant (which is dependent on the ambiance):

(9) Pt=k·Enz0·1‐e−kdecay·t.

For understanding the specific systemic complexities, for example, consider a scenario where *rate enhancement occurs via a catalytic feedback loop*:

dPdt=k1·S−k2·P+fP,

where *f*([*P*]) is a *self-amplification term*. In cases where *positive feedback dominates*, exponential growth can emerge. For example, in radical *chain reactions in peroxidases*, *CYP*, and *cyclooxygenase systems*, the radical reactions could propagate exponentially before termination, giving *burst-phase kinetics* where product formation appears *sigmoidal* but follows *exponential pregrowth*.

For a versatile/realistic accounting of real‐time heme–enzymes′ (e.g., peroxidases and CYPs) routine kinetic assays conducted in labs, with minimal variables (which were earlier erroneously plotted with the Michaelis–Menten interpretations/equations), the internal stochasticity surfaces in rates/values obtained. The radical‐mediated reactions′ dependence on fluctuating conditions is expressed more directly with an empirically extended biphasic (or multiphasic) equation. In competing reactions leading to product formation scenarios, there could be both slow and faster steps (wherein the outcomes would be contingent upon different extents of the reactive species concentrations and include active site processes too!), and/or there could also be simultaneous product depletions also. So, to account for these complexities in a minimal manner, we assume that two independent reaction pathways contribute to the overall kinetics:

dP1dt=k1Subt−consumption of intermediate,dP2dt=k2Subt−slower phase contribution or depletion.

In this scenario, the total product formed is

Pt=P1t±P2t.

If each phase follows a first‐order saturation

P1t=Ymax·1‐e−k1·t,P2t=Ymin1‐e−k2·t.

The total product formation *Y* is

(10) Y=Ymax·1‐e−k1·t±Ymin·1‐e−k2·t,

where the constant *Y*_max_ represents the contribution from the fast initial phase (saturation level for the faster process, e.g., burst‐phase radical reactions); the constant *Y*_min_ accounts for the slower, longer term phase (slower component, e.g., enzyme‐driven reaction); *k*_1_ is the rate constant of the rapid component (diffusion‐controlled radical reactions); and *k*_2_ is the rate constant of the slow component (step‐wise enzyme‐mediated or other secondary reactions). *Y*_max_ and *Y*_min_ have concentration units (or product units, and their graphical interpretation is in the context of yield vs. rate × asymptotic limit).

Now, for availing substrate‐dependent profiles (akin to the classical Michaelis–Menten plot, where *S* is plotted on the *x*‐axis and product formation rate is plotted on the *y*‐axis), we can consider similar fast and slow reactions as above, which are dependent on substrate (or product formed) to various levels. Then

dPfastdt=k1·Seff.

As *S*_eff_ (substrate concentration effectively available for the above reaction) depletes fast and substrate conversion approaches asymptote due to lowering of DRS, we could write

Seff=S−fS,

where *f*(*S*) represents the *fraction of substrate that remains unavailable*. Let us assume that depletion‐driven effects in biological systems follow an exponential decay, as a heuristic rationale (which would be empirically verified by data fitting below):

fS=e−k1·S,Seff=S1‐e−k1·S.

Substituting for *S*_eff_ in the first equation and taking *Y*_max_ = *k*_1_ · *S*, we get

dPfastdt=Ymax1‐e−k1·S.

Similar treatment for the other slow (or higher substrate concentration‐dependent) process and considering *Y*_min_ = *k*_2_ · *S*, we get

dPslowdt=Ymin1‐e−k2·S.

Since both reactions affect total product formation, we can write the equation for the murburn fit:

dPdt=dPfast/dt±dPslow/dt,

(11) dPdt=Ymax1‐e−k1·S±Ymin1‐e−k2·S.

In contrast, the Belanger fit, a special case modification of the MM equation/kinetics for substrate inhibition, is given as

(12) v=Vmax∗SKM+S+S2/Ki.

The above theorization assumes that the substrate binds to another space at/near the active site, thereby preventing product release post reaction. To compare the murburn and classical treatments, two figures are presented. The fitting of data (MATLAB code is given in Box 2, Supporting Information) for simpler aqueous phase polymerization chemistry of pyrogallol to purpurogallin condensation mediated by chloroperoxidase and *para*‐nitrophenol hydroxylation by the complex lipid interface–based liver microsomal system (CYP, reductase, cytochrome *b_5_*, etc.) (as analyzed in Citation #10 of [9]) are presented in Figures 6 and 7, respectively. *Y*_max_ quantifies the maximal magnitude of the activation phase, and *Y*_min_ is of the inhibition phase. The biological significance of *Y*_min_ is that the greater its value, the greater the inhibition effect at higher substrate concentration (as it is for CPO). The murburn model is more appealing visually (captures both the rise and drop) and is theoretically more realistic as it is rather unlikely that multiple enzymes of the oxidoreductases family (e.g., peroxidase and P450) have dual binding sites for diverse molecules. Such man‐made molecules have zilch evolutionary significance as potential substrates! However, the above argument need not hold true for routine (nonredox) metabolic enzymes like hexokinase or tryptophan synthase, which could work via the Belanger/MM mechanism.

Figure 6Fitting experimental duplicates to the murburn and Belanger equations in aqueous phase peroxidase reactions. (a) Experimental duplicates of chloroperoxidase mediate electron abstraction from pyrogallol and fit to the murburn biphasic exponential equation. (b) Same data/values fit to the Belanger equation. A visual examination shows that murburn is a better fit in all regions, particularly the descending part (where Belanger fails).

**Figure:**
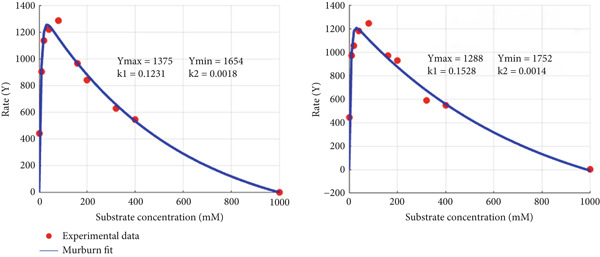
(a)

**Figure:**
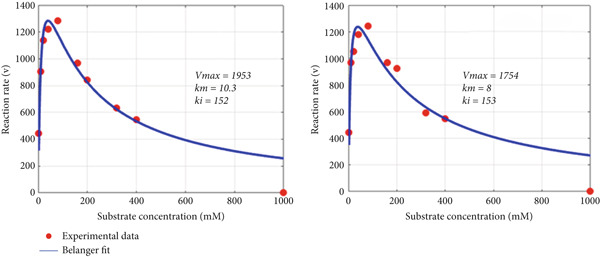
(b)

Figure 7Fitting experimental duplicates to the murburn and Belanger equations in interphase multiprotein reactions (cytochrome P450 microsomes). (a) Experimental duplicates for the CYP2E1 reaction and fit to the murburn biphasic exponential equation. (b) Same data/values fit to the Belanger equation (a special case derivation of the Michaelis–Menten kinetics). A visual examination shows that murburn treatment affords a better fit in all domains of the assay.

**Figure:**
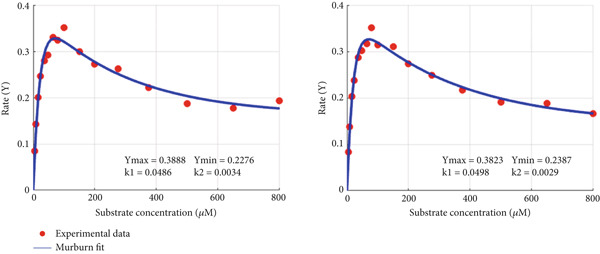
(a)

**Figure:**
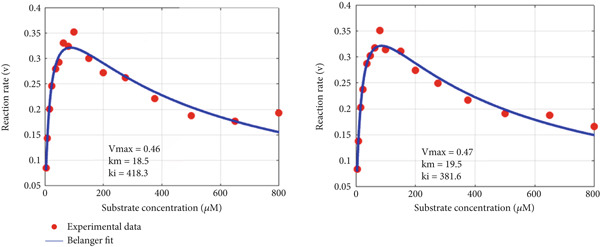
(b)

### 3.4. Heuristics, Empiricism, Exponentiality, and Energy Landscapes in the Murburn Models

Albeit the system being complex, the simple ab initio derivations with reductionist assumptions (or limiting constraints) are useful for explaining experimental realities. The heuristic or semiempirical (theory + fitting) versatile fixes proposed here are akin to using dimensionless numbers (Reynolds′ number as an index for fluid flow regime indication, the Thiele modulus for diffusion–reaction balance in porous catalysts, etc.) and approximations in biochemical engineering treatments commonly employed in bioreactor analysis, as shown in Table 8. Just as Monod made a Michaelis–Menten equivalent for cell growth kinetics (first item in Table 8), Haldane made a derivation similar to the Belanger fit (Relation (12)) for cell growth inhibitions observed at high substrate concentrations. Such extrapolations, although infeasible to derive mathematically from starting considerations, are quite tangible from the point of analogy and quite useful from an application point of view.

**Table 8:** Some popularly accepted relations in biology based on empirical understanding.

**Purpose/name**	**Relation**	**Source/inspiration/comment**
Monod′s cell growth rate	*μ* = *μ*_max_ · [*S*]/(*K*_*s*_ + [*S*])	Michaelis–Menten enzyme kinetics, semitheoretical
Residence time in the reactor	*E*(*t*) = (1/*τ*) · *e* ^−*t*/*τ*^	Probability considerations, empirical
O_2_ mass transfer rate	*K*_*L*_ · *a* = *A* · *N* ^*B*^	System geometry & experimental, empirical

Exponential functions can explain variability in reaction kinetics, and they are not uncommon, with the much‐famous Arrhenius equation (although semitheoretically derivable from the TS theory) as a salient example, correlating the effect of temperature on chemical reaction rates. The exponentiality results in dynamical systems where the rate of change of a quantity is a function of the quantity itself, as is observed in some diffusion‐driven radical reaction systems with pseudofirst‐order kinetics and complex reaction networks. In chemical kinetics textbooks, such schemes are common, as you would have noted such equations for a first‐order derivation (for substrate *A* and product *P*)—*d*[*A*]/dt = −*k* · [*A*], which, when integrated, gives *P*(*t*) = [*A*]_0_ · (1‐e^−*k* *t*.^).

The asymptotic saturation in the time profile curve for product formed in such reactions is similar (although the mathematical origin is different!) to the asymptotic curve observed for the initial “product formation or substrate depletion” rate plotted against substrate concentration. The enzyme is required to maintain/stabilize finite amounts of DRS in the milieu (achieved with a burst), which does the catalysis. The loss of DRS is replenished by enzymes facilitating the equilibrium action (as governed by the classical definition, an enzyme does not alter the position of the equilibrium but only helps it to be attained quicker!). Consequently, the time‐course profile can also demonstrate a flat or multiphasic outcome, quite like the profile of the plot obtained with substrate concentration versus product formation rate. Diffusion‐controlled radical reaction kinetics can involve complex controls and are subject to probabilistic encounters (and this could follow an exponential function). Since murburn systems involve *competition between different pathways and may occur outside the active site*, overall reaction rates can take a *biphasic (or multiphasic form)*, as derived above. Arrhenius‐type exponential dependency on temperature is discussed below. Such ways can give new meaning/modalities and explanations to understanding several physiological signaling/transduction cascades.

With respect to the energy landscapes, traditional enzyme–catalyzed reactions have a well‐defined activation energy (*E*_*a*_) for a given enzyme–substrate conversion barrier, dictated by the active site environment. In murburn systems, electron transfer and radical recombination can follow multiple parallel pathways, each with a different *E*_*a*_. Although the Arrhenius equation assumes the TS theory (where a well‐defined energy barrier must be overcome), murburn ET and catalysis rates may be both diffusion and/or barrier‐limited, depending on the scenario. A Smoluchowski‐like kinetics may result, where the rate is more governed by the probability of radical encounters and not by a fixed energy barrier. Classical enzyme kinetics often shows a sharp Arrhenius dependence at lower temperatures but denaturation at higher temperatures. In murburn systems, the uncertainty in reaction locus means (i) fewer temperature‐sensitive conformational restrictions, (ii) a broader temperature range of activity and possible non‐Arrhenius behavior could result due to radical diffusion effects, (iii) since local ambiance of radicals and redox potentials may fluctuate dynamically, making the activation barriers variable; meaning the reactions can exhibit stochasticity, with rates showing weak or temperature‐independent behavior in some conditions. So we can have a modified Arrhenius‐like empirical expression for murzyme catalysis, as follows. Since murburn systems involve both diffusion‐limited steps and barrier‐limited steps, the rate constant (*k*) can be expressed as a composite equation:

kT=A1·e−Ea1/RT+A2·e−Ea2/RT+DT,

where the first term on the right is the classical transition state pathway and the second term is a radical‐mediated step with a different activation energy. The last one is a diffusion‐controlled term, which may be weakly dependent on temperature. This equation shows that murburn electron transfer/catalysis is a hybrid mechanism involving classical Arrhenius‐driven enzyme reactions, diffusion‐limited radical chemistry, and parallel electron transfer pathways with different energy barriers. As per the equation above, it is predicted that murburn systems should not exhibit a simple linear Arrhenius plot, but rather biphasic or multiphasic behavior could result, reflecting different activation energies at different temperature ranges (Citation #21 of [9]). This is, in fact, the case when data are plotted for peroxidase systems. Since diffusion and radical reactions are often less sensitive to temperature (and at times, even concentration of diverse molecules!) than structured enzyme catalysis, murburn systems may function more uniformly across a wider temperature range, and this could have helped in diverse physiological realms through evolution. In the traditional Marcus electron transfer, the reorganization energy determines the rate. In murburn electron transfer, stochastic radical diffusion and fluctuating redox potentials introduce an additional layer of complexity.

## 4. Summing Up and Future Work

The manuscript presents the first systematic quantitative treatment of murburn ET, catalysis, and inhibition profiles. Besides the derivations offered above, we present a summary of the findings in three heads, as below.

### 4.1. Kinetics of Murzyme Catalysis and Murburn ET

Hemeprotein‐laden peroxisomes, hepatocyte endoplasmic reticulum, and mitochondria are sites of high DRS activity. It must be registered that one‐electron abstractions and halogen atom (e.g., chlorine or iodine) transfers mediated by peroxidases are not the classical Michaelis–Menten‐type reactions but brought out by murburn (DRS) activity. This finding has high relevance in iodinated thyroid hormone synthesis and myeloperoxidase action in physiology. Further, organohalogenics and lignocellulosics are also ecologically cycled by peroxidase action. Biochemistry lab classes can no longer use peroxidase‐based assays as examples of classical enzyme kinetics. They should be correctly addressed as murzyme activity, if not working with active site reactions, at high enzyme and substrate concentrations. The treatments herein reason why the experimental *K*_*M*_ is often lower than the *K*_*d*_ value for the same substrate (as exemplified in cytochrome oxidase and oxygen), when the Michaelis–Menten theory cannot accommodate this apparent falsity. Further, it is inappropriate to report *K*_*M*_ values and IC_50_s for murzymes such as CYPs, as they can show significant variations based on reaction conditions. More meaningful quantitative indices and ways of assessing their functional relevance in physiology need to evolve in the future.

### 4.2. Mechanistic Aspects

**Figure 8:**
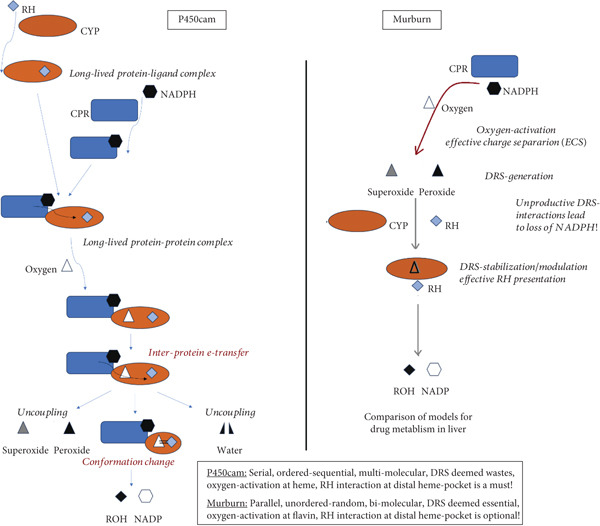
Comparing the two models of CYP‐mediated xenobiotic metabolism.

The murburn model of xenobiotic metabolism is advocated herein (Figure 8), and the premises that seek us to go beyond the classical transition state‐ or active site–based treatments for explaining redox catalysis and BET were discussed, but without “any negative attributes” to the Michaelis–Menten or Marcus treatments, respectively. The equations derived from reductionist/ab initio treatments were used to explain the zeroth‐order dependence of ET rates in some simple and complex redox enzyme systems. That is, the protracted linearity in time‐course profiles with respect to the disappearance of final substrates (e.g., beta‐diketones or sulfides/thioureas or alkenes) in hemeperoxidase mixture or disappearance of NADPH in CYP mixture or consumption of oxygen in mitochondrial system is now mathematically modeled, at a minimal level.

The similarities between hemeperoxidases/mitochondrial reaction systems with the liver microsomal reaction of CYP–CPR are explained holistically considering the involvement of DRS (i.e., murburn concept). (See Table 2 of Citation #23 of [9] for the details of similarities.) Quite simply, these proteins do not have any mandate for evolutionary identification of their substrates. So they just constitutively generate DRS and “burn away” the substrates (whose presence induces greater conversion of NADPH!), producing polar molecules and water, which automatically cleanses the system! Tables 9 and 10 present dozens of comparative analytical points wherein the murburn model renders the P450cam model redundant and obsolete. (Please see Citations #20, #22, and #42 of [9] for greater details.)

**Table 9:** Comparative reasoning of experimental observations in xenobiotic metabolism.

**No.**	**Criterion/observation**	**P450cam (** **X** **)**	**Murburn (** **Y** **)**	**Analytical comments**
1	Presence of xenobiotic (RH) is not obligatory for peroxide detection in the CYP + CPR milieu.	Oxygen activation at the distal pocket of CYP can only occur if RH is not bound via a Type I mechanism in the active site.	Primarily, CPR activates oxygen to give superoxide, which can dismutate to give peroxide.	*X* becomes redundant! Agrees with *Y*.
2	In several reaction mixtures, controls lacking substrates give higher peroxide the test reaction (with RH) in most time frames, and peroxide concentration varies in time.	Uncoupling results due to poor substrate binding and reaction. As CYP and RH do not react with peroxide, their concentration should increase over time.	Agreeable with both scenarios and all time frames, as DROS dynamics differ in various conditions/RH. CPR can deplete peroxide.	*X* becomes redundant! Agrees with *Y*.
3	Taking CPR + NADPH alone in a pure reconstituted system gives peroxide in the milieu.	CPR does not react with oxygen and only serves to transfer electrons to CYP via protein–protein interactions.	Production of peroxide and its dynamics in time are expected with CPR.	*X* becomes redundant! Agrees with *Y*.
4	Taking CPR separately in a dialysis membrane and CYP in free solution does not stop ET to CYP, and a specific hydroxylated product is formed.	Direct protein–protein complexation is required for ET and product hydroxylation.	ET from CPR occurs via DRS, which is used by CYP to generate the hydroxylating agent.	*X* becomes redundant! Agrees with *Y*.
5	*K*_*M*_ values for substrate–CYP interaction vary significantly across labs.	The binding and reaction thereof are specific for a substrate and CYP; *K*_*M*_ should be constant.	The interaction of CYP and RH and DROS has “uncertainty” elements.	Disagrees with *X*. Agrees with *Y*.
6	Stoichiometry (component:NADPH:ROH or NADPH:DROS) varies in time.	There is little rationale for varying stoichiometry under the P450cam scheme, as it follows a deterministic ratio.	DRS–CYP–CPR–RH interaction dynamics evolve in time.	Disagrees with *X*. Agrees with *Y*.
7	Linear disappearance of NADPH.	Expected to show substrate binding–based kinetics.	Accommodates the zeroth‐order linear decay, owing to radical reactions.	Agrees with *Y*.
8	The disappearance of the primary hydroxylated product is seen, along with secondary product formation.	Hydroxylated products do not have affinity at the CYP active site.	A product can undergo secondary oxidations in the milieu, as binding is not critical.	Agrees with *Y*.
9	Several CYPs (also peroxidases) show lower product formation at higher substrates.	Binding‐based explanation seeks a secondary binding site blocking the exit of the product formed.	The milieu could have multiple interactions involving DRS, which could give rise to a lowered primary product in the milieu.	*Y* is more likely.
10	CYPs have high substrate diversity.	CYPs can open up to accommodate the diverse molecules to take them within the bonding distance of Fe–O species.	Substrates could bind outside, on the surface, too. This presents multiple loci for binding‐based selection.	*Y* probable; *X* not!
11	Inclusion of low amounts of SOD does not inhibit reaction, but high amounts do; also, HRP (but not Hb/Mb) with a hydrophobic segment critically affects substrate hydroxylation and increases NADPH consumption.	DROSs are seen as wasteful products, not affecting the reaction outcomes.	DRS interactions are integral to the reaction scheme. Removal of superoxide by SOD/HRP pulls the reaction to the right! HRP can use both superoxide and peroxide.	Explained by *Y*; *X* fails. (Protein–protein binding–based mechanism discredited.)
13	Inclusion of vitamin E and fatty acyl derivative of vitamin C inhibits substrate hydroxylation, whereas Trolox (soluble derivative of Vit. E) and Vit. C do not.	DROS dynamics are inconsequential to reaction outcome.	Lipid‐soluble DROS quencher in the vicinity of CYP–CPR critically affects reaction outcome.	Explained by *Y*; *X* fails.
14	Intramolecular kinetic isotope effects (KIEs) for aliphatic hydroxylation are large for CYPs. This implies that the substrate is free to rotate and orient itself with respect to the reactive intermediate.	A tightly bound substrate is needed for CYP to receive electrons from CPR (for increasing the redox potential) and effective positioning with Fe–O species.	DRS‐mediated catalysis would show high KIE.	Explained by *Y*; *X* fails.
15	Cytochrome *b*_5_ does not give an unidirectional increase in overall reaction rates.	More cytochrome *b*_5_ should give more CPR–Cyt. *b*_5_ and more Cyt. *b*_5_–CYP ETs.	Cytochrome *b*_5_ acts as an electron sponge/buffer. The effects can therefore be bidirectional.	Explained by *Y*; *X* fails.
16	At a constant CYP concentration, increasing CPR gives a hyperbolic curve for product formation; at constant CPR concentration, increasing CYP gives a sigmoid curve, with a much greater rate at higher CYP concentrations.	We would expect symmetric effects as the CYP–CPR complexed pair is supposed to govern the overall catalysis.	Small amounts of DRS generated from CPR are better stabilized and utilized by higher concentrations of CYPs, explaining physiology.	Explained by *Y*; *X* fails.
17	ET and hydroxylations can be obtained with pure DROS and nonconventional redox partners/systems.	Specific binding and e‐tunneling are supposed to govern the outcome.	Intermediation of DRS makes the overall process nonspecific at the core.	Explained by *Y*; *X* fails.
18	Low levels of redox‐active ions, small molecules, and enzymes can inhibit/alter ET by CPR and hydroxylations in the CYP + CPR mixture.	Since the chemistry occurs via deterministic protein bindings, the system is expected to be rugged.	The system seeks the intermediary roles of DRS, which can be easily perturbed by small amounts of redox‐active species.	Explained by *Y*; *X* falls short.
19	Mutations and mechanism‐based inactivations of surface residues of CYPs affect substrate selectivity/specificity and overall activity.	Substrate interacts with CYP at the heme distal pocket.	Substrate can bind or interact with CYP at the surface; this could affect activity.	Explained by *Y*; *X* fails.
20	A small topological or moiety change could significantly alter substrate activity.	Substrate interacts and binds at the active site through the reaction cycle.	The DRS interaction is primarily affected by the reaction center of the substrate.	Explained by *Y*; *X* falls short.
21	Atypical kinetics, mixed inhibition and activation profiles by the same molecule, unpredictable drug–drug interactions.	The overall mechanistic scheme is highly ordered and deterministic.	The reaction scheme is probabilistic, and the components can interact via multiple modes and various loci.	Explained by *Y*; *X* fails.
22	Idiosyncratic and hormetic dose responses.	More ligand should concomitantly give greater outcomes, without individual differences.	Stabilization of DRS at lower concentrations and milieu‐specific manner.	Explained by *Y*; *X* fails.
23	CYPs can efficiently hydroxylate with stabilized superoxide, without CPR.	CYP + CPR binding is essential for e‐relay. DRS is not a part of this scheme.	CYP–RH and CYP–CPR interactions are mediated by DRS.	Explained by *Y*; *X* fails.
24	CYPs show hydroxylations usually more at the energetically favorable carbon, not necessarily the greater accessible carbon.	Since the reactions occur at the active site, spatial constraints should be more important.	Since the attack is essentially by DRS, the reactivity is more important than spatial restrictions.	Explained by *Y*; *X* falls short.

**Table 10:** Reasoning of structure, distribution and theoretical aspects.

**No.**	**Aspects/components**	**P450cam (** **X** **)**	**Murburn (** **Y** **)**	**Analytical comments**
1	Unique/bulky promiscuous CPR; hundreds of diverse CYPs of varying topologies	CYP and CPR must be complex effectively for ET	A unique CPR can relay electrons to diverse CYPs via superoxide	*Y* explains, and *X* fails
2	CPR:CYP is found at 1:10–1:100 ratios	A 1:1 ratio of complexation is minimally expected	Low CPR is expected, as higher CPR would give greater DRS, leading to collateral damage	*Y* explains, and *X* fails
3	N‐terms of proteins are not required for activity	Many advocates root for a TMS‐based interaction between CYP–CPR in the lipid membrane for ET	TMS enhances proximity by embedding in the lipid membrane, and some TMS may have DRS‐modulating residues	*Y* explains, and *X* fails
4	Unique promiscuous cytochrome *b*_5_	Cytochrome *b*_5_ is supposed to serve as an ET relay agent via CPR–Cyt. *b*_5_ and Cyt. *b*_5_–CYP complexations	Cytochrome *b*_5_ serves as an electron sponge/buffer in the lipid membrane. As protein–protein complexations are not sought, promiscuity results	*Y* explains, and *X* fails
5	Type I binding spectrum not observed with most substrates for liver CYPs	Protracted/efficient Type I binding is essential for CYP to elevate its redox potential so as to receive electrons from CPR	High‐fidelity binding of substrate is not sought at the heme center	*Y* explains, and *X* fails
6	Crystal structures of most CYPs show substrates bound away from hemeFe	The substrate moiety should be about 3 Å away from the heme center for direct attack	As the reaction is mediated by DRS in multiple loci, the exact binding locus is not a great issue	*Y* explains, and *X* fails
7	All CYPs′ proximal cysteine ligands are solvent‐accessible	The wiring within proteins concept seeks a secure of e‐losses by exposed redox–active protein structures	Fe could get reduced via superoxide from proximal or distal manner, enhancing the probabilistic scope for Fe–DRS interactions	*Y* explains, and *X* fails
8	Cytochrome *b*_5_ has solvent accessible heme	Residues of cytochrome *b*_5_ should be conserved, and heme should be protected	Since Cyt. *b_5_* is an e‐sponge, it does not need to have conserved residues, and effecting sponging is better with exposed heme	*Y* explains, and *X* fails
9	Some substrates of CYPs are larger than the deep‐seated active sites with narrow entry channels	The active site mechanism solicits facile entry and exit of substrate and reaction product	This model requires the heme distal pocket as a hydrophobic site for prolonging the life of DRS, which emanates from narrow channels	*Y* explains, and *X* fails
10	Most crystal structures′ active site topologies of CYPs do not explain substrate selectivity or product specificity	The active site should have complementarity with substrates for efficacy	The distal heme pocket must be hydrophobic so as to deny ample protons, enabling DRS interaction with substrate in the vicinity	*Y* explains, and *X* fails
11	Substrate‐bound and substrate‐free crystal structures do not show significant differences. Yet the same CYPs may also show different X‐ray crystal structures!	Substrate binding is key to catalysis. Some CYPs need to undergo significant conformational changes. Structures need to be consistent	Hydrophobic protein crystallization with ample substrates can complex it at many places; crystallization depends on protocols	Data supports *Y*
12	CYPs with similar active sites may yet show different substrate preferences	Since the active site is the deciding criterion, we would expect similarity of substrates	The CYP′s surface topology may decide which molecules can bind and avail DRS for reaction	Data supports *Y*
13	Why is CYP3A4 so much more active than the rest?	CYP3A4 has a large active site and, therefore, can accommodate more/diverse substrates	CYP3A4 has diverse channels connecting to several surface crypts (where xenobiotics can bind!), making it more effective	Data supports *Y*
14	Why do some CYPs need Cyt. *b*_5_?	No explanation	The distal heme pocket may be closed or inaccessible, necessitating greater e‐sponging (e.g., CYP2E1)	Data supports *Y*
15	Molecular dynamics	Expects CYPs to alter conformations to interact with RH	Can explain outcomes with given protein structures, as is	*X* is more fastidious than *Y*
16	Diffusion kinetics	Expects the bulky CYPs/CPRs and Cyt. *b*_5_ to be hypermobile	Lipids are needed to localize DRS in its dimension	*Y* explains, and *X* fails
17	Oxygen′s role and formation of DROS	Oxygen must stay bound at CYP′s active site throughout the reaction cycle	Oxygen is free to move around and generate the catalytically important DRS	*Y* explains, and *X* fails
18	Bizarre small values of *K*_*M*_, IC_50_, and *K*_*i*_	Mathematical derivations dictate a strict adherence; for example, *K*_*M*_ cannot be smaller than *K*_*d*_!	Experimentally determined *K*_*M*_ is actually a function of DRS interaction with substrate. So *K*_*M*_ could be smaller than *K*_*d*_!	*Y* explains, and *X* fails
19	Loss of NADPH equivalents (formation of water at heme of CYP?!)	Water could be formed at the heme center to explain the loss of NADPH equivalents. (No evidence or logic for this!)	Loss of NADPH equivalents occurs through loss of DRS via their own interactions or via CPR activity	*Y* explains, and *X* fails
20	Reports of multiple catalytic species	Catalysis occurs via Compound I, formed at the distal pocket	Multiple DRS can get stabilized at the heme center, explaining various observations	*Y* explains, and *X* falls short
21	CYPs can kinetically differentiate between R and S isomers of certain substrates, and yet the preferred isomer is not hydroxylated enantioselectively	The active site of CYP is constrained (access points and Fe–O site). We should therefore expect enantioselective outcomes (e.g., CYP2D6–bufuralol)	The reaction need not be at the heme center. Therefore, enantioselectivity may not be seen	Most CYP reactions are explained by *Y*
22	In silico probing of certain shows great binding at the active site, but this is not matched in reality, as the molecule may not serve as a substrate	Application of the lock and key concept should give corresponding outcomes	The molecule may bind in the pocket in silico, but in real scenarios, its access into the site may be challenged by narrow channels and diffusion limits	*Y* explains, and *X* falls short
23	Some large substrates show (in silico) surface binding to CYPs, and they are converted efficiently	No contextual explanations	Surface binding is the natural modality for most organic xenobiotics	*Y* explains, and *X* fails
24	The larger picture is that most CYP expressions are not inducible by their respective substrates	If the structure of a molecule has an evolutionary connection to function, we would expect inducibility	The xenobiotics pose little scope for evolutionary memory; therefore, there is no “rifle taking aim to fire” but a shotgun pellet type disposition	*Y* explains, and *X* fails

### 4.3. Other Aspects

The heuristic/empirical relations (primarily derived from ordinary differential equations) were also used to fit experimentally derived data for substrate inhibitions, also in simple and complex systems. Therefore, the murburn concept now has a mathematical foundation, which can offer molecular–macroscopic explanations for a bevy of fundamental metabolic and physiological outcomes [7, 8]. It is quite sensible to imagine a nonactive site mechanism for xenobiotics, as they do not present any evolutionary memory for a gene to capture their molecular topography. It would only be natural then for the metabolism to evolve by a generic format, which is presented in murburn.

### 4.4. Future Scope of the Work

A simple analysis of Figure 3 shows that while the disappearance of the substrate NADPH is practically linear, the formation of product peroxide is rather unpredictable (and this is more than evident in Figure 2 for the peroxidatic formation of products from diverse substrates)! Such nuanced and real facts cannot be side‐tracked and should be dealt by real mechanism/kinetics treatment, and the murburn theorization affords unprecedented scope for the same.

In future undertakings within the purview of murburn concept, the efforts shall be to arrive at simple *mathematical correlations* that synthesize/amalgamate *physical* diffusion principles (Brownian motion), electrostatics (Poisson–Boltzmann distributions), nonequilibrium thermodynamics (entropy production and steady‐state fluxes), and *chemical* radical–mediated redox chemistry, delocalized ET, reaction–diffusion coupling, and weak low‐affinity binding, to explain diverse fundamental *biological* problems in bioenergetics, metabolism, physiology, and evolution. In conjunction with AI‐based protocols, it would add new whorls to our understanding of life and could mainstream several borderline ideas and observations, earlier discarded as “artifacts.” We shall soon make available the results of using an advanced murburn theorization paradigm to model neuronal signal transmission and also attempt to simulate/predict murburn hormetic dose responses.

**Nomenclature**
BROS: bound reactive oxygen species
CMPT: classical membrane pump theory
CYP: cytochrome P450
CPR: CYP reductase
DROS: diffusible reactive oxygen species
KIE: kinetic isotope effect
LHC: light‐harvesting complex
PCHEMS: powering, coherence, homeostasis, electromechanical, and sensing–response activities
PDT: photodynamic therapy
PTM: posttranslational modification

## Ethics Statement

The authors have nothing to report.

## Consent

The authors have nothing to report.

## Disclosure

An earlier/preliminary preprint version of this work has been published [42].

## Conflicts of Interest

The authors declare no conflicts of interest.

## Author Contributions

KMM wrote the paper and made the mathematical deductions and logical argumentations. DAG, PMS, and SD contributed to Figure 3 and Table 3.

## Funding

No funding was received for this manuscript

## Supporting information


**Supporting Information** Additional supporting information can be found online in the Supporting Information section. The MATLAB codes have given as supporting information.

## Data Availability

The data that supports the findings of this study are available in the supporting information of this article.
