# Supplementary material for: Quantitative Treatments for Explaining the Mechanism and Kinetics of Catalytic Electron Transfers in Murburn Processes, Particularly Involving Heme Enzymes Like (Per)oxidases and P450s
Source: Biomed Res Int. 2026 Jan 8;2026:3079294. doi: 10.1155/bmri/3079294 (PMC12780854; doi:10.1155/bmri/3079294)
Supplement: Supplementary file 1 — Supporting Information Additional supporting information can be found online in the Supporting Information section. The MATLAB codes have given as supporting information. [file BMRI-2026-3079294-s001.docx]

**SUPPLEMENTARY INFORMATION; Manoj et al., *Quantitative treatments…***

***Box 1: MATLAB code used to generate Figure 6.***

% Time settings

t = linspace(0, 100, 1000); % time vector

% Parameters

kg = 1.0; % baseline ET rate (background)

kComp = 2.0; % competition rate constant

kReact = 3.0; % reaction rate constant

lambda = 0.1; % decay constant for DRS (in biphasic case)

DRS0 = 1.0; % initial [DRS]

% Initial condition

ET0 = 0;

% --- Case (i): Linear accumulation with only kg ---

ET_case1 = ET0 + kg .* t;

% --- Case (ii): Biphasic ET with time-dependent [DRS] = DRS0 * exp(-lambda * t) ---

ET_case2 = ET0 + kg .* t + ((kComp + kReact) .* DRS0 ./lambda) .* (1 - exp(-lambda .* t));

% --- Case (iii): Constant non-zero [DRS] ---

DRS_const = 1.0;

keff = kg + (kComp + kReact) * DRS_const;

ET_case3 = ET0 + keff .* t;

% --- Plotting ---

figure('Color', 'w'); hold on;

plot(t, ET_case1, 'b--', 'LineWidth', 2);

plot(t, ET_case2, 'r-', 'LineWidth', 2);

plot(t, ET_case3, 'g:', 'LineWidth', 2);

xlabel('Time (a.u.)', 'FontSize', 12);

ylabel('[ET](t)', 'FontSize', 12);

legend({'Case (i): Constant kg only', ...

'Case (ii): Biphasic [DRS](t)', ...

'Case (iii): Constant [DRS]'}, ...

'Location', 'NorthWest', 'FontSize', 11);

grid on;

set(gca, 'FontSize', 12);

box on;

***Box 2: The MATLAB code used to generate top-left panel of Figure 7.***

% Data

S = [2, 10, 20, 40, 80, 160, 200, 320, 400, 1000];

Y = [442, 905, 1136, 1220, 1284, 968, 842, 631, 547, 3];

% Define the biphasic equation

biphasic = @(params, S) params(1) * (1 - exp(-params(3) * S)) - ...

params(2) * (1 - exp(-params(4) * S));

% Initial parameter guess: [Ymax, Ymin, k1, k2]

initial_params = [1300, 1900, 1, 0.01];

% Fit the model using nonlinear least squares

fit_params = lsqcurvefit(biphasic, initial_params, S, Y);

% Extract parameters

Ymax = fit_params(1);

Ymin = fit_params(2);

k1 = fit_params(3);

k2 = fit_params(4);

% Display the results

fprintf('Fitted Parameters:\nYmax = %.4f\nYmin = %.4f\nk1 = %.4f\nk2 = %.4f\n', ...

Ymax, Ymin, k1, k2);

% Generate fitted curve

S_fit = linspace(0, 1000, 100);

Y_fit = biphasic(fit_params, S_fit);

% Plot results

figure;

scatter(S, Y, 'ro', 'filled'); % Experimental data points

hold on;

plot(S_fit, Y_fit, 'b-', 'LineWidth', 2); % Fitted curve

xlabel('Substrate Concentration (mM)');

ylabel('Rate (Y)');

legend('Experimental Data', 'Biphasic Fit');

grid on;

title('Biphasic Fit of Substrate Conversion');

hold off;
